# Supplementary material for: Genome-Wide Identification of the U-Box E3 Ubiquitin Ligase Gene Family in Cabbage (Brassica oleracea var. capitata) and Its Expression Analysis in Response to Cold Stress and Pathogen Infection
Source: Plants (Basel). 2023 Mar 24;12(7):1437. doi: 10.3390/plants12071437 (PMC10097260; doi:10.3390/plants12071437)
Supplement: Supplementary file 1 [file plants-12-01437-s001.zip › plants-2293229-supplementary.pdf]

Genome-wide identification of the U-box E3 ubiquitin ligase gene family in cabbage (*Brassica oleracea* var. *capitata*) and its expression analysis in response to cold stress and pathogen infection

Supplementary materials

Peiwen Wang[1,2], Lin Zhu[1,2], Ziheng Li[1,2], Mozhen Cheng[1,2], Xiuling Chen[1,2], Aoxue Wang[1,2], Chao Wang[1,2] and Xiaoxuan Zhang[1,2,*]

[1]College of Horticulture and Landscape Architecture, Northeast Agricultural University, Harbin, Heilongjiang Province, 150030, China.

[2]Key Laboratory of Biology and Genetic Improvement of Horticultural Crops (Northeast Region), Ministry of Agriculture and Rural Affairs, Northeast Agricultural University, Harbin, Heilongjiang Province, 150030, China.

[*]Corresponding author, Email address: zxx780930@126.com

Contents

Table S1......2

Table S2......3

Table S3......16

Table S4......17

Table S5......18

Table S6......19

Table S7......21

Figure S1......23

Figure S2......24

Table S1. Characteristics of *BoPUB* genes in cabbage.

Gene name	Gene ID[a]	Group	Chromosome	Genomic position	CDS[b]	AA[c]	MW[d]	pI[e]	Subcellular localization[f]	Orthologs[g]
BoPUB1	Bol005035	A	C07	38049040-38050311	1272	424	47.58	8.27	nucl:6,cyto:5,chlo:2,vacu:1	XP_013594275.1
BoPUB2	Bol005289	C	C03	43163200-43166241	2292	764	84.28	5.91	nucl:6,cyto:6,cysk:1,golg:1	XP_013628730.1
BoPUB3	Bol005722	A	C08	82316-83647	1332	444	48.32	7.44	cyto:5,nucl:4,chlo:1,mito:1,plas:1,vacu:1,E.R.:1	XP_013612569.1
BoPUB4	Bol007008	E	C08	21666213-21669713	2400	800	88.07	5.34	cyto:7,nucl:6,chlo:1	XP_013604080.1
BoPUB5	Bol007270	A	C01	36294678-36296310	1278	426	47.18	7.25	cyto:8,chlo:2,nucl:2,extr:1,cysk:1	XP_013586402.1
BoPUB6	Bol007667	F	C05	32165369-32167119	1389	463	51.31	8.48	nucl:4,cyto:4,chlo:3,plas:3	XP_013587195.1
BoPUB7	Bol007668	E	C05	32161132-32163836	1146	382	43.77	7.5	chlo:7,mito:4,nucl:1,plas:1,cysk:1	XP_013583587.1
BoPUB8	Bol008036	D	C02	41702893-41704551	1659	553	61.12	6.52	chlo:5,mito:3,nucl:2,plas:2,golg:2	XP_013613640.1
BoPUB9	Bol008152	F	C01	38471230-38472813	1356	452	50.39	8.62	nucl:5,cyto:4,chlo:3,plas:1,golg:1	XP_013620323.1
BoPUB10	Bol008441	C	C03	43797474-43800768	2301	767	85.22	5.08	nucl:11.5,cyto_nucl:7.5,cyto:2.5	XP_013629612.1
BoPUB11	Bol008579	B	C03	44740354-44742513	2160	720	78.71	6.78	chlo:8,mito:3,nucl:2,cysk:1	XP_013629163.1
BoPUB12	Bol009126	A	C04	8207638-8208912	1275	425	47.53	8.71	nucl:6,cyto:4,chlo:2,plas:1,vacu:1	XP_013634337.1
BoPUB13	Bol009241	E	C03	42826034-42829000	2397	799	91	6.09	nucl:6,cyto:4,vacu:2,chlo:1,golg:1	XP_013628398.1
BoPUB14	Bol010350	A	C05	30543476-30545255	1404	468	52.48	7.19	cyto:8,nucl:4,chlo:1,cysk:1	XP_013587093.1
BoPUB15	Bol010460	C	C02	11448388-11451770	2358	786	86.7	5.27	nucl:8,cyto:2,chlo:1,plas:1,cysk:1,golg:1	XP_013621358.1
BoPUB16	Bol011695	A	C04	3917811-3919031	1221	407	45.17	8.49	cyto:6,cysk:5,chlo:2,nucl:1	XP_013634504.1
BoPUB17	Bol012315	C	C07	23724580-23726780	1791	597	66.02	7.3	chlo:6,cyto:3,nucl:2,plas:1,E.R.:1,pero:1	XP_013598353.1
BoPUB18	Bol013021	E	C03	54964336-54966942	1164	388	44.01	4.68	nucl:8,cyto:5,cysk_plas:1	XP_013624944.1
BoPUB19	Bol015885	A	C03	30604113-30605372	1260	420	46.96	8.82	nucl:7,chlo:6,extr:1	XP_013622159.1
BoPUB20	Bol016165	C	C06	4511321-4514149	1860	620	68.01	8.33	E.R.:3.5,E.R._plas:3.5,nucl:3,plas:2.5,cyto:2,mito:2,pero:1	XP_013588277.1
BoPUB21	Bol017004	E	C07	37382487-37385659	2379	793	89.55	6.74	chlo:7,nucl:4,cyto:1,E.R.:1,cysk:1	XP_013599111.1
BoPUB22	Bol018091	A	C05	23579947-23581212	1266	422	45.97	7.53	chlo:8,cyto:5,nucl:1	XP_013586218.1
BoPUB23	Bol018774	B	C01	15040202-15042653	1986	662	71.71	4.85	plas:5,vacu:3,nucl:2,E.R.:2,cyto:1,extr:1	XP_013603034.1
BoPUB24	Bol019224	C	C09	4934468-4937484	2214	738	80.66	4.89	chlo:10,cyto:3,cysk_plas:1	XP_013611968.1
BoPUB25	Bol019726	F	C09	31567100-31568692	1377	459	50.66	8.84	chlo:6,cyto:5,nucl:1,plas:1,golg:1	XP_013609559.1
BoPUB26	Bol021231	E	C02	3528313-3533241	3126	1042	117.86	5.35	chlo:5,nucl:3,E.R.:3,mito:1,plas:1,pero:1	XP_013620771.1
BoPUB27	Bol021760	E	C04	40486202-40489850	2502	834	92.43	6.58	chlo:12,nucl:1,mito:1	XP_013637165.1
BoPUB28	Bol021972	A	C01	18150442-18151785	1344	448	48.69	7.25	cyto:5,nucl:3,vacu:2,E.R.:2,chlo:1,plas:1	XP_013604271.1
BoPUB29	Bol022718	E	C07	7979725-7989744	2010	670	75.38	8.99	cyto:6,chlo:3,nucl:2,cysk:2,mito:1	XP_013595801.1
BoPUB30	Bol022877	A	C03	23114355-23115629	1275	425	46.9	9.32	vacu:9,plas:3,cyto:1,extr:1	XP_013626064.1
BoPUB31	Bol023451	A	C08	927641-928912	1272	424	45.96	6.4	cyto:10,nucl:2,chlo:1,cysk:1	XP_013600441.1
BoPUB32	Bol023972	E	C06	7790036-7793449	3198	1066	118.1	4.97	nucl:4.5,cysk_nucl:3,cyto:2,plas:2,chlo:1,mito:1,extr:1,vacu:1,E.R.:1	XP_013592594.1
BoPUB33	Bol024918	D	C03	39891535-39893145	1611	537	58.4	7.37	nucl:5,mito:4,chlo:3,cyto:1,cysk:1	XP_013622416.1
BoPUB34	Bol025043	A	C08	27739954-27741261	1308	436	49.03	8.74	nucl:9,cyto:5	XP_013600640.1
BoPUB35	Bol026052	E	C03	5985275-5988678	2367	789	88.4	8.64	chlo:10,nucl:2,cyto:1,plas:1	XP_013629141.1
BoPUB36	Bol027262	E	C04	20160334-20165996	1797	599	64.77	6.67	nucl:4,E.R.:4,cyto:2,chlo:1,mito:1,plas:1,vacu:1	XP_013630998.1
BoPUB37	Bol027639	E	C06	1710724-1713541	2430	810	88.9	5.35	cyto:6,chlo:3,nucl:3,plas:1,vacu:1	XP_013591529.1
BoPUB38	Bol028412	D	C01	7866975-7868078	1104	368	40.15	6.91	cyto:5.5,cyto_nucl:5.5,nucl:4.5,chlo:3,mito:1	XP_013617834.1
BoPUB39	Bol028991	D	C01	1265612-1268155	2259	753	84.31	6.05	nucl:5,cyto:4,chlo:2,mito:1,plas:1,cysk:1	XP_013613296.1
BoPUB40	Bol029355	E	C09	17336879-17341604	1530	510	57.61	8.88	nucl:8,cyto:3,pero:2,vacu:1	XP_013615869.1
BoPUB41	Bol029357	E	C09	17383689-17387122	2034	678	76.5	8.88	nucl:4,E.R.:4,vacu:2,chlo:1,cyto:1,plas:1,golg:1	XP_013615869.1
BoPUB42	Bol030454	E	C09	33424858-33429958	3114	1038	117.23	5.41	nucl:6,chlo:3,cyto:2,plas:1,E.R.:1,pero:1	XP_013611998.1
BoPUB43	Bol030961	A	C01	31316399-31317652	1254	418	46.05	9.85	nucl:9,chlo:5	XP_013639712.1
BoPUB44	Bol031005	A	C01	30733021-30734274	1254	418	45.75	7.09	chlo:6,cyto:4,nucl:3,plas:1	XP_013594317.1
BoPUB45	Bol033248	B	C04	32033767-32036354	1965	655	72.01	5.4	E.R.:3.5,E.R._plas:3.5,nucl:3,plas:2.5,golg:2,chlo:1,cyto:1,mito:1	XP_013637553.1
BoPUB46	Bol034488	F	C03	3645792-3651503	3060	1020	111.34	6.91	E.R.:4,cyto:3,nucl:2,vacu:2,chlo:1,mito:1,plas:1	XP_013623822.1
BoPUB47	Bol034489	F	C03	3652498-3654286	1395	465	51.44	5.09	cyto:9,chlo:4,vacu:1	XP_013628277.1
BoPUB48	Bol034491	F	C03	3671228-3673202	1419	473	52.61	6.3	nucl:5,chlo:3,cyto:3,plas:3	XP_013628281.1
BoPUB49	Bol036591	B	C01	22029097-22031136	2040	680	75.06	7.84	chlo:8,nucl:2.5,mito:2,cysk_nucl:2,cyto:1	XP_013605655.1
BoPUB50	Bol036718	B	C05	25576732-25578822	2091	697	76.69	7.33	chlo:8,nucl:2,mito:2,plas:1,E.R.:1	XP_013638039.1
BoPUB51	Bol037760	A	C04	36502645-36503883	1239	413	46.31	8.43	cyto:7,chlo:2,cysk:2,nucl:1,plas:1,vacu:1	XP_013633566.1
BoPUB52	Bol037877	B	C04	35662808-35664799	1992	664	73.52	7.16	chlo:8,nucl:3,mito:2,cyto:1	XP_013636377.1
BoPUB53	Bol037990	E	C05	4401778-4406478	1896	632	70.77	8.72	nucl:6,cyto:6,chlo:1,vacu:1	XP_013607202.1
BoPUB54	Bol039769	A	C03	8905416-8906642	1227	409	45.45	8.48	cyto:7,chlo:3,nucl:2,cysk:2	XP_013623595.1
BoPUB55	Bol040674	E	C05	39733-41926	921	307	34.45	6.7	cyto:7,nucl:4,chlo:1,extr:1,cysk:1	XP_013584685.1
BoPUB56	Bol040675	E	C05	43012-45429	1308	436	49.72	4.73	nucl:9,cyto:4,cysk:1	XP_013586791.1
BoPUB57	Bol040676	E	C05	45765-48401	930	310	35.35	4.8	cyto:7,nucl:4,cysk:2,vacu:1	XP_013586789.1
BoPUB58	Bol041392	B	C03	35763128-35765315	1971	657	71.17	4.89	plas:7,vacu:3,E.R.:2,cyto:1,extr:1	XP_013629415.1
BoPUB59	Bol041756	C	C07	16043256-16046166	2136	712	78.49	6.22	cyto:8,nucl:5,plas:1	XP_013597568.1
BoPUB60	Bol041877	B	C07	17530961-17533096	2136	712	77.35	6.17	chlo:5,cyto:3,mito:3,nucl:2,golg:1	XP_013599003.1
BoPUB61	Bol044051	E	C09	39181374-39183068	1098	366	39.72	4.69	nucl:10,chlo:3,cyto:1	XP_013613332.1
BoPUB62	Bol044176	C	C04	24020183-24022584	2094	698	76.84	6.28	nucl:5,mito:3.5,chlo:3,cyto_mito:2.5,plas:1,cysk:1	XP_013637194.1
BoPUB63	Bol044185	F	C04	23729237-23732001	1245	415	47.31	8.52	nucl:7,cyto:5,chlo:1,vacu:1	XP_013631296.1
BoPUB64	Bol045384	B	C08	29129169-29134639	2694	898	98.04	5.29	chlo:5,nucl:2.5,cyto_nucl:2.5,plas:2,E.R.:2,cyto:1.5,mito:1	XP_013604382.1
BoPUB65	Bol045811	C	C08	34875756-34878494	2433	811	87.94	5.24	chlo:6,nucl:2,cyto:2,plas:1,E.R.:1,pero:1,golg:1	XP_013605017.1

[a]Accession number from the *Brassica oleracea* var. *capitata*_446_v1.0 genome database.

[b]Length of coding sequence (bp).

[c]Number of amino acids in the protein sequence.

[d]Protein molecular weight (kDa).

[e]Theoretical isoelectric point (pH).

[f]Subcellular localization of BoPUB proteins was predicted by the WoLF PSORT software. The values represent the accuracy probability of subcellular localization prediction. Nucl, nucleus; Cyto, cytosol; Mito, mitochondria; Chlo, chloroplast; E.R, endoplasmic reticulum; Cysk, cytoskeleton; Plas, plasma membrane; Vacu, tonoplast; Extr, extracellular; Golg, golgi bodies; Pero, peroxisome.

[g]BoPUB orthologs in wild cabbage (*Brassica oleracea* var. *oleracea*).

Table S2. Amino acid sequence of BoPUB proteins.

Gene name	Amino acid sequence
BoPUB1	MDQEIDIPCFFLCPISLDIMKDPVIVSTGITYDRDSIEKWLFTGKNNSCPVTKQVITETD<br>LTPNHTLRRLIQSWCTLNASYGIERIATPKPLISKSEIEKLIKDSSASYQNQVKCLKRLR<br>QIVSENPTNKRCLEAAEIPEFLAKIVSNSVDTSNDLCQSNMLENRFDSSRSLMDEALSIL<br>YHLDTSEEARKSLLNNKKGTNLVMTLTKIMQRGNYESRAYAAFLLKKILEVADPMQIILL<br>ERELFNEVVQILNDQISHKATTSAMQILVIICPWGRNRHKAVAAGAVSMIIELLMDETSS<br>SERRNSEMAMVVLDMLCQCAEGRAEFLNHGAAIAVVSKKILRVSQITSERAVRVLLSIGR<br>FCATPCLLQEMLQLGVVAKMCLVLQVSCGNKTKEKAKELLKLHARVWRESPCVPRNLYAS<br>FPA*
BoPUB2	MDLSELEENLFAASDAKLHRDMCKELSAVYCNVLSIFPSLEEARPRSKSGIQALCSLHIA<br>LEKAKNILQHCSECSKLYLAITGEAVLLKFEKTKSALIDSLRRVEDIVPSSIGSQILDIV<br>GELEHTKFLLDPSEKEVGDSIIALLQQGKKFDNGTDNAELEIFHQAATKLSITSSRSALA<br>ERRALKKLIDKARAEEDKRKESIVAYLLHLMRKYSKLFRSEPVDENDSQCSPPCSPTGNE<br>DRPHAFGRQLSKFGSVNNSRKPGQMMPTPPEELRCPISLRLMCDPVIIASGQTYERVCIE<br>KWLSDGHNSCPKTQQQLPHLSLTPNYCVKGLIASWCEQNGITVPTEPPQSLDLNYWRLAL<br>SDSDSRSCTPKDAKLVPLEESSTIGSEQQQHKEDVTVADVDVEETSAAINVLEQYQDILA<br>ILDKEEDLGEKCKVVENVRLLLKDDEEARILMGANGFVEAFLRFLESAVHENDVAAQETG<br>AMALFNLAVNNNRNKELMLTSGVIPLLEKMITFPHSQGPATALYLNLSCLEKAKPVIGSS<br>QAVPFFVKLLLKGETQCKLDALHALYNLSTHSPNIPTLLSCNIIKTLQVLASTGDHLWIE<br>KSLAVLINLASSQEGKEEMISSQGMISTLATVLDAGDTVEQEQAVSCLVILCTGSEQCIQ<br>MVLQEGVIPSLVSISVNGSSRGRDKSQKLLMLFREQRQREQASPNKDDAPRKSLSAPLPM<br>SVPAQASAQPGSEGKPLFKSTSARRTMTRAFSFFWKKSYSIHR*
BoPUB3	MPMFQPFKGGGFDGHVIDLHTAVKDGVLGGGDGGEVKTAAVENELDLKTMITNLELPETP<br>SVFICPISLEPMQDPVTLCTGQTYERSNILKWLSLGHRTCPTTMQELWDDAVTPNKTLHQ<br>LIHAWFSQKYVMMKKRSEDVQGRVIEIVGALRKAKGKEKVHALSELKGVVMAHEIAKKSV<br>VDEGGVFVISSLLTPFTSHAVGSEAVAILVNLELDAVSKAGLMQPARVSLMVDMLNDGSI<br>ETKINCARLIGRLVEERGFRAELVSSHSLLVGLMRLVKDRRRRSGVSPALALLKSISVHK<br>QVRSLMVSVEAVPQLVDVLPCLGPECLESALYVLDSLCWDNEGVTALKDSVNTIPNTVRL<br>LMRVSETCTAYSVSILWSVCRLASRECSSLAVELGLAAKLLLVIQSGCDPALKQRSAELL<br>KLCSLHYSDTMFISKCKLAPTIQ*
BoPUB4	MDGSESDDSSHFERGVDHIYEAFICPLTKEVMHDPVTLENGRTFEREAIEKWFKKCQDSN<br>KSPSCPITSQELSSADVSPSIALRNTIEEWRWRNDAAKLDVARQALFLGNDESDVLQALM<br>NVRQICRSIRSNRQGVRNSQLVRMIIDVLKSNSHKVRYKALQTLQVVVEGDEESKALLAE<br>GDTVRTLVKFLSHEPSKGKEAAVSLLFELSKSEALCEKIGSVNGALILLVGLTSSNSVNV<br>SIVDKADRTLENMERSEEVVRQMASYGRLQPLLGKILKGSPETKLSMTTFLGELSLNNDT<br>KVYVAQTVGSSLVDLMRSGDMPQREAALRALNNISSYEGSAKLLISIGILPPLIKDLFYV<br>GPNNLPIRLKEVSATILANIVNIGYDFDKATLVSDNRVENLLYLISNTGPSIQCKLLEVL<br>VGLTTCQKTVIHVVSAIKTSGAIISLVQFVEVKENEDLRLASVKLLHNLSPFMSEELADA<br>LRGTAGQLDSLVAIISEKIPISEEQAAAAGLLAELPERDLALTREMLTVGAFEKIISKVV<br>GIRQGEIKGMRFERNFLEGLVRILSRITFAFSNETLQTRAITFCREYNVASLFIHLIQSN<br>GQDTIQMVSAMALENLSLESINLSHMPDLPPPSCGSIFSCMSKPPVITGLCKIHHGVCSL<br>RETFCLVEGGAVEKLVALLDHENDKVVEASLAALSSLLEDGLDVEKGVKILDEADGIRHV<br>LNVLTENRTEKLTRRAVWLVERILRIEDIAREVAEQPNVSASLVDAFQNGDFRTRQIAEN<br>ALKHIDKIPNFSGIFPTMA*
BoPUB5	MKDPVTAVSGITYDRQSIVQWLEKVPSCPVTKQHLPLDSDLTPNHTLRRLIQHWCDENAT<br>RGVARIPTPRAPPGKLNIAEEIKNLKKFVVEASGREDTLKKLEVLAMEGETNRIMMCEAG<br>VHRSLILFVVKCTLEEEEEGQHLKGHVDESLRLLHLIGVPLNDTRTILIENDQILESLTL<br>ILHQQDFINKAYTVVLLRNLTGNASSHIVERFGAEILKGILGFLKDVVISSFNLTNLSVS<br>ATLQPSSSSVRSKLDRSLVIKRGVTAALMILLESSSWCRNRTILVLDLGAVSQLIDLEISY<br>SGEKRTTELVLGILSRLCCCADGRGEMLTHGGGIAIVTKRVLWVSTAADNRALSILSTLS<br>KFSPENEVVNEMVCVGTVEKLCSVLRVDCGLSLKEKAKEILRDHFDEWKRFPCIDVPLLT<br>KLLSS*

BoPUB6	MAKTDAFDSDPTAIAKAKELKREMKKLLTNIEEEDGLSIRTIDQLQEALSAFKQATMRKM<br>AKSSSLEMLETVSCPEEFRCPLSNELMRDPVVLASGQTYDKLFIQKWLSSGNRTCPKTQQ<br>VLPHTALTPNLLIRDMISKWCKTVGLETTTYQSSLVDEEKAVTRSDREIFNSLLCKLSSS<br>NLQDQKSAAKELRRLTKKGTEFRALFGESSDNITRLVNPLLLHGSTLNQDEQLQEDVITT<br>LLNVSIHDDSNKKLVCENANVIPLLIDALRRGTVATRSNAAATVFTLSALDSNKALIGKS<br>GILKPLIDLLEEGNPSAIKDAAAAIFTLCIAHENRSRAVKDGAVRVLGKKISEGLNVDEL<br>LAILAMLVTHWKAVEELGELGGVTWLLRITRESECKRNKENAIVILHTICFSDRTKWKEI<br>KEEESSHGTITKLAREGTSRAQRKANGILDRLRKAMNLTHTA*
BoPUB7	MALITRREVIAHNVLTKKRKEIVERASQLDVVVSNRLARSEPRRQVEKKKYYLLELRSLF<br>SSILFWRDSTHAPPEEEKFSVRSSRKKRESLRFDRIVLEMATGAVSPMAAKQAERLKEDG<br>NNCFKKERFGAAIDAYTEAITLSPKVPVYWTNRALCHMKRKDWTRVEEDCRKAIQLDHDS<br>VKAHYMLGLALLQKEEYADGVKALQRALDFGRGANPTGYMVEEIWEELSKAKYMEWELLS<br>AGRSWELNSLKETCVAALNQQRALDMSRTEESSEEDYSSHTDQLKALDRVFEKAAEEDKP<br>TEVPGYLCCNITLEIFRDPVISPSGVTYERAAILEHINKVGKFDPITREELDPSNLVPNL<br>AIKEAVAAYLEKHVWAYKTGC*
BoPUB8	MGKSGRLRWNPFSHRSSSSRQEQNQQQQQPPIEFICPISKTIMSDPVVVSSGQTFERVCV<br>QVCRDLDFIPKLNDLNDSLPDFSTVIPNRNMKSTIDAWCDSVGVERPHPPDYSAVETNLR<br>HQMPPSSSSETEIRVSEQELLRAVADRAPVIYHHADSDLMGRRKPTGDFNSTSSDESVIV<br>AQSPFTPLPLTTRPACFSPSSSSSEIEFSAAAAANSPEEEEIYNKLKSSEVFDQEQGLIM<br>MRKMTRTRDEARVSLCSSRILALLKLMIVSRYSLVQTNSLASLVNLSLEKTNKLTIVRLG<br>FIPLLIDVLKSGSKEAQEHAAGSIFSLSLEDDNKMPIGVLGALQPLLHALRAAESDRTRH<br>DAGLALYHLSLNQTNRLKLVRLGAVQVLFSLVRSGESASRALLVICNLACCSEGRSAMLD<br>ANAVGILVGKLREEEERRGEESRSSAAARENCVAALFALSHESLRFKGLAKEARLVEVLK<br>EVEERGTERAREKAKKILQLMRERVPEEEEDDGEGTVDWDRVILDSNGSIRSRFRDGGGG<br>RNRMVTQNSSGF*
BoPUB9	MAKTEFFDSDPTVIAKTKELKREMKKLLRTIEEDDDLSVQTIDQLQETLSDFKRATIKKM<br>AKSSSLEMLETVSCPEEFRCPLSKELMRDPVVLASGQTYDKLFIQKWLSSGNRTCPKTQQ<br>VLSHTGLTPNLLISDMISKWCKKVGVETMNQYQSNTKSNREVLNSLLCKVSSSNLQDQKS<br>AAKELRLLTRKGTVFRALFGESSDQITRLVNPLLSLNQDEHLQEDVITTLLNISIHDDNN<br>KKLVCENPNVIPLLIDALRRGTVSTRSNAAAAIFTLSALDSNKSLIGKSGVLKPLIDLLE<br>EGNPLAIKDAAAAIFTLCIAHENRSRAVKDGAVRVLGEKISDGLYVDELLAILAMLVTHW<br>KAVEELGELGGVSWLLKITRESECKRNKENAIVILHTVCFSDRTKWKEIKEEESSYGTIT<br>KLAREGTSRAQRKANGILDRLRKAMNLTHTA*
BoPUB10	MDVNEVEESFFAPGDAKLHGEMCNALSVIYCKIMSVFPSLEAARPRSKSGIQALCSLHVV<br>LEKVKNILRHCTESSKLYLAITGDSVVLKFEKAKTSLIDSLRRVEDIVQQSIGSQILEII<br>MELENTQFSLDPSEKEVGDQIIGLLQQGGNFESSSDNNELEIFHQAATRLGITSSRAALS<br>ERRCLKKLIERARMEDDKRKESIVAYLLHLMRKYSKLFRSEIWDDNDSQGSNSLPCSPTI<br>EGSLDDPPGRAFDRQLSKLSSFNFRPCNNNRRSVQMSVPPDELRCPISLQLMYDPVIIAS<br>GQTYERLCIEKWFSDGHNTCPKTQQELSHLCLTPNYCVKALISSWCEQNGVQVPDGPPES<br>LDLNYWRLALSVSESGKSVGSCKFKDVKVVPLEESGTIKEESSCELEYQEAEVTLVERCT<br>DLLTTLSGVDTLRKKCRVVEQMRVLLKDDEEARILMGENGCVEALLHFLGAALSEKNDSA<br>QKVGAMALFNLAVDNNRNKELMLVSGIIPLLEEMLCNPHSHGSVTALYLNLSCLEDAKPV<br>IGSSLAVPFMVNLLWTETETQCKVDALHALYHLSTYPPNIPCLLSADIVNALQSLTVSDD<br>QRWTEKSLAVLLNLVLNEAGKEEMVSVPGLVSNLATILDTGEANEQEQAVSLLLILCKYS<br>EMCSQMVLQEGVIPSLVSISVNGTQRGRERAQKLLTLFRELRQRDQTHHTTTEEHVEVVC<br>PEEGGFSVAAASVTESKPQCKSASRKKMGRAFSFLWKSKSFSVYQC*

BoPUB11	MATAAIFSSLRRRRSPSLEAFLSPVDLSGLPLVQTLAAISTEIISSFSGTRFSFQRRNAR<br>SLIRKIEIFLVLFESLAESRWGSTPSSSSSSSSTALLCLKELYLLLYRSKILLDYCAHSS<br>KLWLLLQTQSISGYFHDLNQEISTLLDVFPVNDLNLSDDVREQIELLQSQSRRSRLYIDN<br>NDESLRRTFYSFLDGFEHGEIPNTLALRSFFVEKLGIKDSKSCRSEIEFLEEQIANHDGD<br>VEPTGSVINGFVAITRYSRFLLFGFEEEWRIKNNNNPKKKSKGDGNDGFITVPKDFVCPI<br>SLDLMTDPVIISTGQTYDRTSIARWIEEGHCTCPKTGQMLVDSRIVPNRALKNLIVQWCA<br>ASGVSYESEFVTDSPTNEGFVSGLPTKAAVEANKATVSILIEYLADGSEAAQTVAAREIR<br>LLAKTGKENREFIAEAGAIPHLRRLLKSENAVAQENSVTAMLNLSIYEKNKSRIMEEEDC<br>LEAIVSVLVSGLTVEAQENAAATLFSLSAVHEYKKRIAMADQCVEALASLLQNGTPRGKK<br>DAVTALYNLSTHPENCSRMIEGGGVSSLVGALKNEGVAEEAAGALALLVRQSLGAEAIGK<br>EESAVTGLMVMMRCGTPRGKENAVAALLELCRRGGAAVAERVLRAPAIAGVLQTLLFTGT<br>KRARRKAASLARVFQRREHAAMRAGGYGFVGDVNGNRDGGNFTTDVSVPMSISISVPVL*
BoPUB12	MDQEIEIPSFFLCPISLDIMKDPVIVSTGITYDRNSIEKWLFTGKENSCPVTKQVITETD<br>LTPNHTLRRLIQSWCSLNASHGIETIPTPKPPISKSEIERLIKDSSSSHKNQVKCLKRLR<br>QIASENNTNKRCLEAAEVPEFLANIISNLVGTSSSLNDMSNMLENRFDSSRSLMDEALSL<br>LYHLNASETARKSLLNNKKGTNLVKTLTKIMQRGIYESRAYATLLLKKILEVADPMQIIL<br>LERELFTEVVQILHDQISHKATKSAMQILMITCPWGRNRHKAVEAGAASMIIELLLDESF<br>SSERRNVEMAMVVLDVLCQCAEGRAEFLNHGAAIAVVSKKILRVSQITSERAVRVLLSVG<br>RFCATPCLLQEMLQLGVVAKLCLVLQASCGNKTKEKAKELLKLHARVWRESPCVPRNLYA<br>SYPA*
BoPUB13	MEETQNQVLEEEAKGSTTVEKVYIAVGNDLQEGFKTIDWALKKWDNIPISIVLLHLCSIS<br>QDFVYTPFGKLPASSVSEEKLQVLRKYEDQKINKLLSKYVTFCKKLQVKAELHKVEKEDD<br>SIQVLILDLITKLRITKLVMGITFMRSSSSWKSKSAISGSFHIYQNKPDFCEFYIICGGK<br>MVLLKRENDANNNIRSWIGKMFHDPGRNLDRSSNGSDDPAASGSPWDKNLQEMEIYFQQL<br>LSLNLEEDDEDNVQEEEDEDGGDEVALDVLQHLNVGEKLEYVRRKVNEAKLMIDENMREV<br>KVNAERSDKAEWAISLCNCRIEELEAGIKEETERREKLQGTLDSDRECIEQTKNDVEKGK<br>AKLVSLGELREELSSKVETMREAKQRAEAELERVALEKGEMIMEIEKLKNQRDVFNRRIE<br>FCKEKGLLDSKEEEVKCGYREYVAEDIRLATESYSDRLRLKSGGNWTNVYRGRIKHTTMA<br>VKVIGDRLSDEEFAAKVKLLNEIRHPNLVAIAGFSSERPKCILFEYMHSGNLRDNLFTSQ<br>RKTRRSKILKWHDRIRIAHQVCSGLGFLHSVKPKPIVHGRLTPSKILLDRNLVAKITGFG<br>LVMHSDQSDTKPDVMAFGVLLLHLLTGRNWPGLLKAMSMNQASILRDLDQTAGKWPLELA<br>KEFGALAVKCSSVNRGGNMDFSTKEIMEELGKIMEKAKEFRTKGGYEEATNSKNDEADPN<br>DIPSVFICPILQEVMKNPHIAADGFSYELEAIEEWLSMGHDTSPMTNLRLDYQVLTPNHT<br>LRALIQAWHSKTAAQASS*
BoPUB14	MDHEEEEIEIPNYFICPISLEIMKDPVTTVSGITYDRQSIVQWLEKVLSCPVTKQPLPLD<br>SDLTPNHMLRRLIQHWCVENATRGVVRIPTPRAPPGKPNLIEEIKNLKKFGQEALGKEDT<br>LKKLEVLAMEGERNRRLMCEEGVHKSLILFVVKYTREEEEEEGQRRIKGKLDESLRLLHL<br>IGVPLNEARTILIENERILESLTLVLNQQDFMNKAYTIVLLRNLTENTSSHIVERLSADI<br>FKGIIGFLKDVVSSSNRVNPSMCATVQPPNSRVRNKAPSKLDHSVVIKQAVTAALMILLE<br>TSSWSRNRTILVDLGAVSELIELEISSTSEKRTTELVLGILSRLCCCANGRAEFLAHRGG<br>IAIVTKRLLRVSAAADDRAISILSSVSKYSPESEVVEEMASVSTVKKLCSVLSIDCSLSL<br>KEKAKEILRDHIDEWKKFPCIDVALVTKLLSSSPKDLLTEYYSRVGV*
BoPUB15	MDVTELEENLFAASDAKLHGDMCKELSGVLCKVLSIFPSLEGARPRSKSGIQALCSLHIA<br>LEKAKNILQHCSESSKLYLAITGDAVLLKFEKAKVSLINSLKRVEDIVPSSIGSQILEIV<br>GELENTRFLLDPSEKEVGDQIIALLQQGKKSDNCNDNTELEIFHKAATRLSITSSRVALA<br>ERRALKKLIDRARAEEDKRKESIVAYLLHLMRKCSKLFRSEILDENDSSQGSAPCSPTVQ<br>EDNGSVHGFGRQLSRFGSMNFKPINSPRSGQMPVPPEELRCPISLQLMCDPVIIASGQTY<br>ERVCIEKWFSDGHNTCPKTQQQLPHLSLTPNNCVKGLIASWCEQNGTQIPSGPPESLDLD<br>YWTLALSGSECTNSKSVNSIGSCNMKGIQNVTTIVEQQYTEESFVSDNDDDDDKEDSDMN<br>VLERYQDLLAILNEEEDLDKKGKVVEKVRLLLKDDEEARIFMGANGFVEALLRFLGSAVD<br>ENNAAAQERGAMALFNLAVNNNRNKELMLTSGVIPLLEKMISSSESHGPATALYLNLSCL<br>EEAKPVIGSSQAVPFLVHILQGEAENQCKLDALHAIYNLSTYPPNISALLSSNIIKTLQG<br>LLASTVEHLWIDKSLAILLNLASSQQGKDEAVSSQGMISSLATVLDMGDTTQQEQAVSCL<br>LILCNGRESCIQMVLQEGVIPSLVSISVNGTTRGREKSQKLLMLFREQRQQRDQPSPKRD<br>EEPPSQKEAPRKSMSAPMYVHESSAQPSDSGPEFEPRVLSKSMSRRKSLARPFSFFWKKS<br>YSTRQ*

BoPUB16	MDEEIEIPPFFICPISLEIMKDPVIVSTGITYDRDSIEKWLFSAKKNSCPVTKQDITDAD
	LTPNHTLRRLIQSWCTLNASHGVERIPTPRPPISKSEIEKLLKDSASSHQNLAKCLKRLR
	HIVSENASNKRCLEAAGVPEFLATVVSNKDSSMILTDEALSLLYHLDTSETGLKNLLNSK
	KGNDIVNSLMKIMQRGIYESRAYATFLLKNILEVADPMQIMTLKPAVFTEVVQILDDRIS
	HKATKSALHILVNVCPWGRNRHKAVEAGVISMIIELLMDESFSSDRRGPEMAMVVLDLLC
	QCAEGRAEFLNHGAAIAVVCKKILRVSQTASDRAVRVLFSVGRFCATPALLNEMLQLGVV
	AKLCLVLQVSCGSKTKDKAKELLKLHARAWKESPCLTRNMILAYPS*
BoPUB17	MAGVIIPPASLLKRIAEIAEIPLNAGVFKKDCTDLTRRVSLLTHLIEEIKDSNQIDSAAS
	SSSENDWWSDLVVALEASKRLLSSAVRFQARDSSDVAAKRISFQFQCVTWKLEKALSNLP
	YDLYDISDEVREQVELARSQLRREMQRYGSLNSNKFSSALFELMERDVKIKVEEEKDESV
	AETLHLAEEKKQLTKSPSISLDFYLSKDADSERLDKMVTKNTDESSKLTVPVDFLCPVSL
	ELMKDPVIVSTGQTYERAYIQRWIDCWNLTCPKTQQKLQNFTLTPNYVLRSLISRWCSEH
	NIEQPANGRCGDMSMIRSLVRSLSNRSLEEHRNAVSEIRSMSKRSTDNRIMIAEAGAIPV
	LVNLLTSEDVATQENAITCLLNLSIYDNNKELIMFAGAVTSIVQVLRAGTMEARENAAAT
	LFSLSLADENKIIIGGSGAIPALVDLLENGTPRGKKDAATALFNLCIYQGNKGRAVRAGI
	VPALVKMLSETSSHRMMVDEALTILSVLASNLDAKSAMVKANTLPALIGILQTGQGRNRE
	NAAAILLSLCKRDNERLVLIGRLGAVVPLMELSKNGTERGQRKAASLLELLRKACQ*
BoPUB18	MAEALIDDPVYVAVSTDVSESRLTLTWALRHLQPKKLYLLHVHQPISINPTSSGLEQSEI
	DAIQESELTSSYEILLKYRDICVVEGILEQDVDISYSLANNVGEGIVELIYENNIKKLIM
	GAAADSHNSEDMVNITSRKFDYVTKHAPHCCKIWLVGNGNLIHTREGRFDRRGSPHPSSE
	SLTSLQGLDSALVPYEEAVRGENDVSHALSSPEDQSARGFETMYYEQQRRGLEIEERRIK
	AEEDLRAEIENMKGIQKELEEQLYIDCPRQFEMFQKERDEAMKTTVELLRLLNLDNSESA
	SHSPPSSFQRSVSNEPPPYFLCPITQEVMREPSVAADGHTYEAEALREWLDNGHDTSPMT
	NLKLAHRNLVPNHPLRSAIHEWLQEHS*
BoPUB19	MRKDDLYITVPSFFRCPISLDVMKSPVSLCTGVTYDRASIQRWLDGGNNTCPATMQILQN
	KDFIPNRTLQRLIEIWSDSVRRRASVESAELAAPTRDEIADAIDRVKVEKEERFDREVLA
	KILRFARESDDNRGFLAGKDDFVRLLVDLMSQIDFKTTSAAKLLIVQEAVKILSMIQSKI
	SDRRRFSNLVLNNGRDRLTTIVYFYKTGNVELKIDCAGLLEFIAVDSESKVSIAERDGLV
	VELMKSISRDSDSSLIEATLSCLIAISSPRRVKLNLIREKLIKDLTKLLTDPTTASVSVT
	EKCLKLLESLASTKEGRSEICGGDGECLKTVVRKLMKVSTAATEHAVTVLWSVSYLFKEE
	KALEAVTSTNGVTKILLLLQSNCSPAVRRMLTDLLKVFKVNSRSCLSAYDTKTTHIMPF*
BoPUB20	MAGVAVSPASLLDVIAEIAEISANSGVFKKDCADLARRVCLLTHLVEEIRDSPQTPTEEE
	SDASSSLVSCECDWWSDLVVGLQAAKRLLSAATCFQARESSEGAAKRISFQFQCVTWKLE
	KALGNLPYDRYDISDEVREHVELARLQLRRAMQRYGSLNSKKFSTALSEPMERDSSSKVT
	EKLECIPETIHSNIPSSDEKKLESPPRRKSSSASLAFFLSKDADIERLEKAITKTNDDDS
	KKSNNLTIPEDFLCPISLELMKDPVIVSTGQTYERSYIQRWIDCGNLRCPKTQQKLGNFA
	LTPNYVLRSLISQWCTKHNIEQPGGYTKNCEGEVSAIRALVRKLSTRSLQDRRTALSEIR
	SLSKRSSDNRILIAEAGAIPVLVKLLTSEDVETQEKAVTCVLNLSIYESNKELIMLAGAV
	TSIVQVLRAGTEEAKENAAATLFSLSLADENKIIIGASGAIPALVNLLENGSVRGKKDAA
	TALFNLCIYEGNKGRAVRAGVVNPLVKMLSDTSSHRMVTEALTILSVLAGNKDAKTAILR
	ANAVPTLIALLQKDQPRNRENAAAILFAICKRDKEKLILIGKLGAVVPLMELSRDGTERA
	KRKANSLLELLRNSSQKLC*

BoPUB21	MREGALIVAVAIKGNNSKTKGVIRWALQEFASQEHVVFKLLHVQPRDSMSVSTSRKGSTT<br>TVYKKDVDRKTREMLHPSSSMFAHREVQLDMMVLESDDVADAISKAVQDHGISELVIGAS<br>SSSIIFSWKLKRSNLSSRIADVTPRFCTVHVISKGKLVNVRKSDVDIETSIADDRSESQF<br>SSSSQSGSVSSTSSHQFSSTSLLYQRVQALSTVNQKVGTNMGTTKSIDTHHSRAASLDVD<br>EPNQRGYYRTNSSLIRYKESDIHSRRSSLTEEGSSSGCYSDPTSCSSQMNKDFELEKLKI<br>ELRHIKGMYAVAQSEVLDATKKMQDLNQRRSEEATRLKNLTIREEYAEEAVEMERERQEE<br>AENEAELVRESVERETEERLEAEARAEEVRKEKQRLEDALEGGPLQRQQYMKFEWEDIVQ<br>ATSSFSDELKIGTGGYGSVYRCNLHHATVAVKVLHSDKSSLTKQFHQELEILSKIRHPHL<br>LLLLGACPERGSLVYEYMHNGNLEERLMKRRPNTDAPQQPLLWFERFRIAWEIASALYFL<br>HTNEPRPIVHRDLKPANILLDQNNVSKIGDVGLSKMVNLDPSHASTVFNETGPVGTFFYI<br>DPEYQRTGVVTPESDIYAFGIILLQLATARSAMGLAHSVEKALRDQTGNFSEILDETAGD<br>WPVKEAKEMVMIGLRCAEMRKRDRPYLGKEILPVLERLKDVASDARNMFSETVSTHHNHA<br>PSHFHCPITKDVMENPCVASDGYTYEKRAIKECLEKNHKSPMTDSPFPNQTLLPNHSLLF<br>AIKEWRSSHAIT*
BoPUB22	MPRSLEPLDLGIQIPYHFRCPISLELMRDPVTVCTGQTYDRTSIESWVSTGTDTTCPVTR<br>APLSDFTLIPNHTLRRLIQEWCVANRSNGVERIPTPKQPADPTSVRALLSQASAACGTHV<br>SVRSRAAALRRLRAFARDSEKNRVLIAAHNAKEILIRILFSDESPELVSEALALLAMFPM<br>TEPGQCESIISDAGRVGFLTRLLFDSSIETRVNAAALIEMVVTGSKTADIKGTVLTSECI<br>FEGVIDLLRNPTVTSSPRRALKIGVKALFALCLAKNTRRIAVSSGAPEILIDRLAAGLDR<br>CDAERALATVELLCRTAEGCAAFGEHALTVPLLVKTILRVSDRATEYAAGALLALCTAEE<br>RWRDEAVAAGVVVQLLLMVQSECTERAKRKAQKLLKLLRDSWPDYSFANSDDFACSEVVP<br>F*
BoPUB23	MEEEKGAVAQSLIDVVNEISSVSEYRTTVKKLCSNLARRLKLLVPMFEEIRESNEPINED<br>TLKTLVSLKEAMSSAKNYLKFCSQGSKIYLVMEREQVTSKLLEVSVQLEQSLSKIPYEDL<br>DISDEVREQVELVLSQFRRAKGRVDASDDELYEDLQSLCIKSSDVDDYQPTLQRVAKKLH<br>LMEIPDLAQESVALHEMVASSGGDAGESIEEMAMVLKLIKDFVQIETNNSEDQSAGVNSS<br>SNGQTSTAASHKIPVIPDDFRCPISLEMMRDPVIVSTGQTYERTCIDKWIEAGHSTCPKT<br>QQALTSTTLTPNYVLRSLIAQWCEANDIEPPKPPSSLRPRKVSSFSSPAEANKIEDLMWR<br>LAYGNPEDQRSAAGEIRLLAKRNADNRVAIAEAGAIPLLVGLLATPDSRIQEHSVTALLN<br>LSICENNKGAIVSAGAIPGIVQVLKKGSMEARENAAATLFSLSVIDENKVTIGALGAIPP<br>LVVLLNEGTQRGKKDAATALFNLCIYQGNKGKAIRAGVIPTLTRLLTEPGSGMVDEALAI<br>LAILSSHPEGKAIIGSSDAVPSLVEFIRTGSPRNRENAAAVLVHLCSVDPQHLVEAQKLG<br>LMGPLIDLAGNGTDRGKRKAAQLLERISRLAEQQKETAAQAQNTEEAEPTHSASTTEAAD<br>T*
BoPUB24	MEVSWLRVLLDNISSYLSISSMDTLYSNPAHKYYTRGEDIAKLLQPVLENLVGSDASPSE<br>LLNNGFEELSQYVDELREQFQSWEPLFSRIFYVLQIESLATKLRESSLEVFQLLKHCEQH<br>LPADLVSPSFEDCIELVKLVGRDEVSYTIDLALVDQKEGNGPTSEVLVKIAESVGLRSNQ<br>EILIEGVVLASLKENAELTENNTEAEFIEGLISLITHMHDHLTNIKQSQLGCSVPVPPDF<br>RCPLSLELMTDPVIVTSGQTYERAFIEKWFDMGLMVCPKTRQPLTQTSLTPNFIVKAFIS<br>NWCESNNVNLPDPLELVQSSQPFPLLLEPASDDDDECGSQPLSLVDESERGSSSPMKIGG<br>NGRTESLDATKCEKLRQVLSRSVSAPGIVFELDSKTERNTTPPAPAPRAPADTSVSQARV<br>ETVRRLPSARHFHHPGIIPATIRETGSSSSIESEVKKLIEDLKSSSLEAQREATARIRIL<br>SRNSTDNRIVIARCGAIDSLVNLLYSTDERIQADAVTCLLNLSINDNNKSVIADSGAIEP<br>LIHVLRTGNLEAKENSAATLFSLSVIEENKTKIGEAGAIEPLVDLLGNGSLRGKKDAATA<br>LFNLSIHHENKAKVIEAGAVRYLVELMDPAAGMVEKAVVVLANLATVKEGKVAIGEEGGI<br>PVLVEVVELGSARGKENATAALLQLSMHSQRFCNSIIREGAIPPLVALTKSGTARAKEKA<br>HNLLKYFKSQKQGQRRG*
BoPUB25	MTDSTTTTAADAVTLKREFKKILTEILSYGGESKDHGETGVLMKAIDEANRIINCLREVE<br>PGTDILSPPSPEKVDVPKEFKCTLSKKIMIEPVIIASGRTYEKRYIKEWLKRERTCPISN<br>QVLSHVSLTPNLLVDELITQWCLVNKFERPKSSEEIVTELFTDGIDSLLQRISSPSSVAD<br>QAQAAEELRRQTKRFANVRAFLVSELPDSIKRLLTPLSAVDLNLELQENLITTLFNLSIV<br>EKNKTVIAKNPLVIPLLTKSLKQGTAETRRNSAATLLSLSAIESNRLIIGNSETLKALIG<br>LIGEGGLFTTEAASAVFNICSVSENREKAVSSGLIPVLTNKIKEGSNVAELLALLALLST<br>HNRGVKEMNDLGFISDLLSILRKPSCPVTHENAVVIVFNMCDRNRDRSGRLKVLSEEENQ<br>YGTFTKLAKQGSDRAVRKAEAILKWLKRHGTGKAPQRE*

BoPUB26	MSTSKPQRSPAEIEDIILRKIFYVTLTDSTTAAAAPDPRVVYLEMTAAEILSEGRDLLLS<br>RDLMERVLIDRLSGTFPAAEQPFPYLVGCYRRAHDESRKIQSMKDKNLRSEMEIVTREAK<br>RLAVSYCRIHLANPDMFGSVDDKTIGGVGVGMKKRSVSPLLPLIFSEVGSGSLDMFGGSS<br>SCGGAQSPPGFLDEFFKDSDFDNLDVILKELYEDLRSTVINVSVLGDFQPPLRALKYLVS<br>LPVGAKSLVSHEWWVPRGAYMNGRAMELTSILGPFFHISALPDNTLFKSQPDVGQQCFSQ<br>ALERRPADLLSSFSTIKNFMNILYSGLHDVVMILLKSTDTRECVLQFLAEVINANASRGH<br>IQVDPVSCASSGMFCNLSAVMLRLCEPFLDPHLTKRDKIDPKYVFYGHRLKLSDLTALHA<br>SSEEVSEWIDKDNTAKATDARNENESRLLQSKEATSSSSNASGQNAKSATKYTFISECFF<br>MTARVLNLGLLKALSDFKHLAQDISRGEDNLATLKAMRDQSPSPQLELDITRMEKELELY<br>SQEKLCHEAQLLRDGDFIQRALSFYRLVVVWLVGLVGGFKMPLPSTCPMEFSCMPEHFVE<br>DAMELLIFASRIPKALDGVQLDDFMNFIIMFMASPEYVRNPYLRAKMVEVLNCWMPRSSG<br>SSATSTLFEGHQLSLEYLVRNLLKLYVDIEFTGSHTQFYDKFNIRHNIAELLEYLWQVPS<br>HRNAWRRIAKEEEKGVYLNFLNFLVNDSIYLLDESLNKILEIKKIEAEMSNTAEWEQRPA<br>QERQDRTRLFHSQENIVRIDMKLANEDVTMLAFTSEEITAPFLLPEMVERVANMLNYFLL<br>QLVGPQRKSLSLKDPEKYEFRPRQLLKQIVRIYVNLARGDSENIFPGAISSDGRSYNEQL<br>FNAGADVLRRIGEDGRIIQEFMELGTKAKAAASEAMDAEAALGEIPEEFLDPIQYTLMRD<br>PVILPSSKTTVDRAIIQRHLLSDNHDPFNRAHLTSDMLIPDVELKARIDEFVRSHQSKKR<br>ASGEDSNKERIQTTSSDMLID*
BoPUB27	MALVTPIPAMSERAGSMRFHGITTTSPGSRSSRSSVTEEPVSRLIEEKIFVAVDKHVAKS<br>KSTLVWALQNTGGKKICVVHVHQPSQMIPVMGAKFPVSSVKEEEVKVFREKEREKVHMIL<br>DEYLRICHQRGVRAEKMFIETESIENGIVQLISELGIKKLVMGAAADRHHSKKMTELKSR<br>KAIFVRREAPPLCQIWFTCKGYLIHTREAIDDGESEYVASPRPSISANDLLQALSRPESG<br>SVQRLGSNGSSTEHSERVSNGSLNTTDDEERDFDGSGVGGSATVMSTVDENSGRSSPSNF<br>PDGVDDSFHDKIRQATSEAQSSKREAVAENVRRRKAEKNALDAIKRVKQSETAYSEELKR<br>RKDTETAVAKEKERFVTIKKEQEAVTEEVQTATAQKHTLENQIAEADTTMETLNKKLDIA<br>VKLLQKLKSEREELQSERDRALREAEELRTLATETTSTLQHQLLPHYFTDFSFSEIEEAT<br>NRFDSTLKIGEGGYGSIYVGTLRHTQVAIKILNPKSSQGSVEYQQEVEVLSKTRHPNIIT<br>LIGACPEGWSLVYEYLPDGSLEDRLTCKNNSPPLSWQNRVRIATEICAALVFLHSNKAHS<br>LVHGDLKPANILLDGNLVSKLSDFGTCSLGRSKSASTDLTGTVPYLDPEASSSGELTPKS<br>DVYSFGVILLRLLTGRPALRIANEVKYALDSGSLNNLLDPLAGDWPFVQAEQLARLALRC<br>CESVGENRPDLGTEVWRVLEPMRASSGGSSSFHLGRNEQRIAPPYFICPIFQEVMQDPHV<br>AADGFTYEAEAIRAWLDSGHDTSPMTNAKLSHNSLIPNHALRSAVQEWLQHHC*
BoPUB28	MPMFQPLKRDGLVGFEGGGDGQVLDLDTAVKDGLLGGVNGGGGVVDEKIDLKTMIKELDL<br>QDIPSVFICPISLEPMQDPVTLCTGQTYERSNIHKWFSLGHLTCPTTMQELWDDAVTPNR<br>TLHHLIYTWFSQKYVLMKKRSEDVQGRAIEVLGTLKKAKGQARVHALSELKQIVVAHLMA<br>RKTVVEEGGVSVISSLLGPFTSHAVGSEVVAILVSLDLDSDSKSGLIQPAKVSLIVDMLN<br>DGSNETKVNCVRLIRGLVEEKGFRPELVSSHSLLVGLIRLVKDKRHRNGVSPALGLLKPI<br>SAHKQVRSLMVRVGAVPQLVDILPSLDPDCLESALFVLDALCSDMEGRVAVKDSANTIPY<br>TVKVLMRVTESCTNYALSILWSVCKLAPEECSPLAVEVGLAAKLLLVIQSGCDPALKQRS<br>AELLKLCSLHYSDTMFISKCKLTRTIQ*
BoPUB29	MVEMLTQKGREMSSGGGGPKAEEGELYVAVAVKGIIGDKLGGAGSRRAVRWAVDNLLPKA<br>DRFVMIHVIPPISTIPTPNGERLPLEEVEERLVEMYVRDVKQEYETVFVPFLKMCKSNRT<br>KCQVETLLIEYDDPAKALLRFIYKSGVNSLVMGSFNSNIFTRRAKGPGVPLTVLKYAPET<br>CEVYIVCRDRITTKSMDPLINAAPCTSPKAAATPRRFLKDGAASFHTVQTQTSSDPGESI<br>EVGTRRSTSAKELRLEALSLAIREPETPQSSKASRATVQDVIRRRGGSDIPQLNYSDFDE<br>TTEQKSNIENIVKEQRDSNPPPATSRKSKKVEIEAEVERLKKELQNTVVKYKQACEELFS<br>TQNKVQVLSSECSKDARRVNNAVEKEELHRKTAALEKERYMKAIKEVEAAKALLAREYCQ<br>RQIAEVNALKNYLEKKKVIDQLLGTDQRYRKYTIEEIFIATEGFSPEKVIGEGGYGKVYS<br>CSLDSTPAAVKVVRLDTPEKKQEFLKEPNGLIHTVENAVNKGTLTEMLDKSVTDWPLAET<br>EELARIGLKCAEFRCRDRPDLKEEVIPVLKRLVETANSKIKKERSNLRAPSHYFCPILRE<br>IMEEPEIAADGFTYEKKVILAWLEKHNISPVTRQKLDHFKLTPNNTLRSAIHDWKSRVRF<br>SNAVVNITG*
BoPUB30	MGRDETERYITVPSLFRCPISLDVMRSPVSLCTGVTYDRASIQRWLDGGNNTCPATMQIL<br>RTKDFVPNLTLQRLIKAWSDSVGRRADGSPLRGIPTVEEVNESLRRLSLEKDDEIRLENL<br>SRIVRFAKDSDANREFLWKRKDFAPMLVDIIAAVDGGTRATAKIKLGLLAIMILDTIIKG<br>GKERDRERLSKLMLTDGGGCLAALLLAIRRGNLKTKIESVRVLEMISFDAKSKEIISECD<br>GIVPELIKSISSDTDPSLMEASLSFLITISTSKRVRSKLIAEKAITKIKDILLTETTSVA<br>VTEKSLKLLETLSSKREGRSEICGGDGGRCVEGVVRKLLKVSAAATEHAVTILWCLCYVF<br>REDKKAEETVVRCNGLTKLLVVIQSSCSPVVRQMAKDLIKVLKVNTSASVLSAYETNTTH<br>ITPF*

BoPUB31	MPGNLEPLDLGIQIPYHFRCPISLELMSDPVTVSTGQTYDRTSIESWIATGNTSCPVTRL<br>PLSDFTLIPNHTLRRLIQEWCVANRSSGVERIPTPKQPADPISVRSLLSQASAISGTHVS<br>VRSRAAAIRRLRGLARDSEKNRVLIAGHNAREILVRILFADVDVGVETSSSEVVSESLAL<br>LVMLHMTEGECESISSDPGRVGFMTRLLFDSSIENRVNAAALVEMVLTGSKSTDLKMIIS<br>GSGSVFEGVMDLLRSSVSSRRALKIGIKALFALCLVKQTRHLAISAGAPGTLIDRLAADF<br>DRCDTERGLATVELLCRLPEGCAAFGEHALTVPLLVKTILRVSDRATEYAAGALLALCTA<br>EERCRDEAAAAGLVTQLLLLVQSDCTERAKRKAQMLLKLLRDSWPDDTLVNDDEFGRSEV<br>VPF*
BoPUB32	MEIQTGESSITCPTDVIESLSESIEAANYLLEITQESNGPESTTDLTSIEAGFQGVVKQI<br>GETLQSIPEAIIDEEEYIGVVVQSLSNEMQNANIGDGSKSEILQNIQQKISERRTQEELV<br>SEEQIETDLYPSDPEVSYASYLSESQPDIQSQSTYVSSQRKYGDLSESQMSEIPDIPSQS<br>TIVSSSQRKYGTQSKSQSQVSEIVDIPSQSTYVSSIQRKYGTQSESQSQVSEIPDISIRS<br>TNVSSRQRKFGTLSESLSMLPQVTQFMEPPYQAFICPLTKEVMEDPVTTETGVTCERQAV<br>TEWFDKFGDSDEISCPVTGQKLTTGLSPNLVLKTIIGEWKVRNEAARIKVAHAALSLGGS<br>ESMVIDALRDLQMTCEGKEYNKLKVREAGIIQLLDRYLSYRSKDVRYELLQLLKTLADED<br>TDEGKEMIVNTLAMSCVIKFLGSSHQTVRHAALALLLELSKSQHACKKIGNATGAILMLV<br>TSKYNEESDAFASETADQILKNLEKFPHNIKQMAESGLLEPLLIHLAEGSEETQVVMAAY<br>LVEIDIGHEKKINVAEKACPALLRLVQSENIEARRAAFKALAHISLYHPNKQILVEVGII<br>KIMVEEIFTKRMFSDLMNSRNEAATILANILESGVEHETFEVNTTGHTLGSDYFVYNIIQ<br>MLKNSSPDDLNIDLIRILLSLSKSPRAMATIVSVIKETDASFAMIELINNPHEELGVGAL<br>KLLIALTPFIGHTLSERLCKTRGQPENLIQCPAEANLITEKHAVSAKLLAKLPHQNLTLN<br>LALVNESIVSEILHAIHLIQRSGTRTSRYATDFLEGLVGILVRFTTTLYEPQMMYLAKNH<br>DLTSVFADLLMKTSSDEVQRLSATGLENLSSTTMNLSRPPQVRNTKFMGSLSMPRSFSLR<br>SSKKKQVETCAIHRGVCSAKATFCLVEANAVTKLLACLQSDKTEVVESALSAICTLLDDK<br>VDVEQSLNMLSGMNAVELIINAVKEHKKESLLQKAFWMIDKFLIRGGQRYAFGISQDRML<br>SGMLVSAFHRGDGNTRQMAENILRRLDKMPSFSVYMTERVTSETP*
BoPUB33	MGENGRHRWFSFHHHRSASATSMPQNNPGETPAEFLCPITGFLMSDPVVVASGQTFERIS<br>VQVCRNLGFAPKLHDGSQPDLSTVIPNLAMKSTILSWCNRKKVEHPRPPDYAYVEGVVRA<br>RMDKEPGTGNRVARSEILPPVAENSPSDYDSVMGAIRARSRNSISSSTSLPLHHHRPVTY<br>SSSSEVFAGAENPNPIQSSFSTSDYSSFPPMSEEEEEIYNKMRSSNTVDHEQGLILLRKT<br>TRSSESSRISLCTERLLSLLRSLIVSRYNIVQTNAAASLVNLSLEKPNKLKIVRSGFVPL<br>LIDVLKSGSTEAQEHVIGALFSLAVEEENKMVIGVLGAVEPLLHALRSSESERARQDAAL<br>ALYHLSLIPNNRTRLVRAGAVPMMLSMVRSGESASRILLLLCNLAACSEGKGAMLDGNGV<br>AILVGKLREGGADSEAARENCVGALLTLSVGNMRFRGLASEAGAEEILTEIVESEGGSER<br>LKEKARKILQAMRGGEREFGEGAEAREWNRMLEASGLSRSQFQGGQKEGFAYSSQF*
BoPUB34	MDQEIEIPTFFLCPISLDIMKDPVIVSTGITYDRDSIEKWLFTGKKNTCPVTKQVITETD<br>LTPNHTLRRLIQSWCTLNASHGIERIPTPKPPISKSEIEKLIKDSSSSHQNQVKCLKRLR<br>QIVSENTTNKRCLEAANVPEFLAKIVSNSVDSYNSPSPSLSSSNLNDLCQSNMLENRFDS<br>SRSLMDEALSLLYHLDTSETARKSLLNNKKGTNLVKTLTKIMQRGIYESRAYATFLLKKI<br>LEVADPMQIILLERELFNEVVQILHDQISHKATKSAMQILVIICPWGRNRHKAVEAGAIS<br>MIIELLMDETFSSERRTLEMAMVVLDMLCQCAEGRAEFLNHCAAIAVVSKKILRVSQITS<br>ERAVRVLLSIGRFCATPSLLQDMLQLGVVAKMCLVLQVSCGNKTKEKAKELLKLHARVWR<br>ESPCVPRNLYASYPA*
BoPUB35	MVVMSNKFFESIGGAPAYSMVAVAVKGSVGDAVGGAASRRALRWTVENLLPNVDRLVLVH<br>VMPTVTTIPSPSGSKIPVDELEESVVSMYKRDLNKEYEQVFVPFKKLCGSSKVETLLMEH<br>DDPAKALLKYVSDSEVECLVLGSCSSSFLTRKKGQEMPLRVLREAPETCEIYVICKDRIL<br>TKSTNQLSPDSSSSFRIPQGAEAYTETFSRTRSDRTGLSASSMSSSGRKHSRRPASLPHS<br>NPVSRVFSDAQSSTDIGLVDDEHTRSVIRHSIVSGNKMQLNPGANIKTPKSDVKSEVAQL<br>RKEVETTLSMYKQACEELVHKQTQVKSLSSECIKETRRVITALEKEEMLRKAAAEEKEKH<br>LKAVKEVQEAKSMLAKEFCDRQLAELSALKQSIEKQKVIDQLFLKDGRYRKYTKEEIAAA<br>TDNFSSRKIIGEGGYGKVYKCSLDHTPVALKVLKPDSIEKKEEFLREISVLSQLRHPHVV<br>LLLGACPDNGCLVYEYMENGSLDAHISPKKGKPSLSWFIRFRIIYETACGLAFLHNSKPE<br>PIVHRDLKPGNILLDRNFVSKIGDVGLAKLMSEESPDSVTVYRNSIIAGTLYYMDPEYQR<br>TGTIRPKSDLYAFGIIILQLLTARHPNGLLFCVEDAVKRGCFGDMLDGSVRDWPMAEVEE<br>LARIAIKCSQLKCRDRPDLDTQVLPALKRILESANERLKREQDNVRPPSHYYCPILKEIM<br>EDPYIAADGFTYEGRAIRAWIQHNQNVSPVTKHRLKHCDLTPNHTLKSAIQEWRSRSRLD<br>LSTTLGSF*

BoPUB36	MNCAISGEVPEVPVVSTKSGLLFEKRLIESHISDYGKCPVTGEPLTIDDIVPIKSGKVIK<br>PKLLHTASIPGLLGTFQNEWDGLMLSNFALEQQLHTARQELSHALYQHDSACRVIARLKK<br>ERDEARQLLAEVERHIPAAPEAVTANAPLSNGKRAADDEETGPDAKKLCPGISADIITEL<br>TDCNAALSQKRKKRQIPETLASVDALGRFTQLSSHPLHKTNKPGICSMDILHSKDVIATG<br>GIDATAVIFDRPSGQILSTLTGHSKKVTSVKFVGDSDLVLTASADKTVRVWRDSGDGQYA<br>CGHTLNDHSEEVRAVTVHATNKYFVSASLDSTWCFYDLSSGLCLAKVADDSEKVDYTAAA<br>FHPDGLILGTGTSQSVVKIWDVKSQANVARFDGHTGEVTSISFSENGYFLATAAEDGVKL<br>WDLRKLRNFRSFSSADANSVEFDPSGSYLGIAASDIRLYQTASVKAEWNLIKTLPDLSGT<br>AADVLLWRNKKISAGSSDGSYCDLGAFRVDQRPFSLSLVCYRLLLGMIVQLVWSNASGAL<br>NRSQSGVPRLVLPKDFFAEVGVTIGTEVNRGLMFLQDLACRGSLKQFLMVVNILLTSA*
BoPUB37	MAASCDGSQSDQSSTFEPGIDNIYEAFICPLTKQVMQDPVTLENGQTFEREAIEKWFKEC<br>VQNGKPVLCPITSKQLSITDLSPSIALRDTIEEWRARNDCMKLDIARQSLYLGNSEANIL<br>LALKNVRDICRNIRLIKRRVRNPQLVRLVTDMLKSTSHEVRYKALQTLRVVVEGDEESKA<br>IVAEGDTVRTIVKFLSKEPRRGREAAVSLLFELSKSELLCEKIGSVHGAILLLVGLTSSK<br>SENVSTVEKADLTLTNLERSEENVRQMASNGRLQPLLAKLLGGSLETKASMASYLGELAL<br>NNDVKFNVAQTVGSCLIDLMRSRDMRGAALGALNKISSFEGSAKVLIGTGLLPPLIKDLF<br>YVGPNQLPIRLKEVSATILANIVNIGYEFDKVPVGAHHQTLVSEDVVENLLLLISNTGPE<br>IQGKLLEVLVGLTSCPNSVINVVSAIRNSGAIISLVQFVEVHDNDDLRLAAIKLLHNISP<br>HMSEELASVLRGTVGQLRNLVGIISESTTTITEDQAVAAALLAELPERDWGLTQRLLGDG<br>AFEKIISKIVLIRQGEIRGKRFERTFLEGLVKILARITFALTKEAQAVSFCRENNLASLF<br>LDLLQSYSQDNIQMASAIALENLSLETKNLTVIPELPPPSYCVSIFSCLSKPHVVLGMCK<br>IHQGICSLRDSFCLVEGQAVDKLVDLLDHENDKVVGPALAALSTLLEDGLEVENAVKLIV<br>EADGLTPILNVLLENRTENLRIRAVWMVERILRIEDIARELGEEQNVTAALVDAFQNADF<br>KTKQIAENALRHIDKIPNFSGIFAPTLAK*
BoPUB38	MAFELPNDFRCPISLEIMSDPVIIQSGHTFDRVSIQKWIDSGNRTCPISKLPLSESPSLI<br>PNHALRSLISNFAHVSPEQEDHSHSQSHPSISTLVSRSSSRESRLESLTRLVRLTKRDSS<br>VRRKVTESGAVRAVLDCVDSVDRALQEKSLSLLLNLSLEDDNKVGLVADGVVRRIVAVLR<br>VGSPDCKAVAATLLTSLAVVEVNKATIGSYPDAISALVSLLRHGNDRERKESATALYALC<br>SFPDNRKRVVDCGSVGILVEAADSGMERAVEVLGLLVKCRGGREEMSRVSGFVEVLVNVL<br>RNGSLKGIQYSLFILNCLCCCSREIVEEVKREGVVEICFGLEDNESEKVRRNATNLVHTL<br>LGNPMEA*
BoPUB39	MGITQRIEKLPHSYKVGCKNLFHCFFYFACNNIFGLALFQMHSSMCLELKNLVDRVMRIF<br>PDIEDARPGSSTGIQTLCLINKALEKAKLLLQYCSESSKLYMAVTGEAILSRGCRAKKLL<br>EQSLSDIRSMVPTVLATKITQVVQDLRSTELALESSEEEAGKAVRELMKRSTTSSVSSDE<br>VRDFHYAALKLHLSTPEAIVIERRSLKSLFAKLGECEVNKRKILKYLLCLLKKHEKIIWR<br>DHKENSFTQLQSVNDSVCASDAEAGCSEEEHNATLPEHFKCPLSLTVMYDPVMISSGHTF<br>ERMWIQKWFDEGNESCPKSRRKLDDFTMKPNVAMKEQISKWCSKNGLDVQDPTTKHAKSS<br>HNLDFSIASFGSSLYNLDFSSRDFSSSFSTDPPSYSKGGYFMPMKTIASESGTEVTDSSQ<br>SEVEIEPLSELIKLPWEAQVKTIQDVKKRFENNSRAFQSMLPSKFLEPLVTFLKNAAGTN<br>ANTIKSGLALLLTFLSGNRKAIEALEEDVFETLSVFLGFELLVAEEALNVLEFLSNQPHS<br>LSKITLSSLMKMAESGPEHLQEQAVITLKNLSTSNEICLEMVTLGFVKNLTLFLQQSVFS<br>KQSIIIMRNLCNTEKGRVCITETPSCLASIAELLDSNVPEEQETAISILLQLCVEKIEYC<br>NLVVREGVDIYPSLFLISNNGTEEAKVGASELLRALEEVDFNREEEEEKESSTTTSQVVT<br>PVMHQDPIITTPSPKKSGLFGLSFSILKKKKR*
BoPUB40	MNYFFKKSYLETKPVLDQVPTKAGSDVASPSQSLTIAVALSGSTKSKNVLKWALRKFASD<br>KNVIFKLIHVHPKITSVPTPSGKTVYISEAPEDLVATYRRQMMEETKETLLKQYRKMNRD<br>VTAKIPAYVSDLCTVYVVSKGVYILTKDKLSSDGEINETILRDIGSERIDTSSYSSSLGH<br>ISDATLKSKSLAMSNHKRLEHLPTIVRGVSVHMETSSVDSYGTTSMSSGAAEKASSSLET<br>SRTISWNPPRSYMSSSENITQGEDYFTGKQDTLDKITKLRDEHGHAQEMHALTQVETLNA<br>FLKLDEFEFEDLQLKEQATKGLKEKETQKFEQRRREEREVAQKREAKEKEKLKESSLVAR<br>KLQYQEFTWEEIKTATSSFSEDLKIGKGAYGDVYKCNLRHTIAAVKVLHSPKSNLFKQFD<br>QENVAVKARNLISSAPNQPPSHFLCPLLKDVISEPCVAADGYTYDRRAIEEWMQDHRTSP<br>VTNLPLQNINLLPNHSVYEAIVEWRRNNQ*

BoPUB41	MDYFFRNSYLETKSVLRQVPKKAGSDLASPSQALNVAIAISGSTKSKNVVKWALEKFSSD<br>KNLVFKLIHVHPKITSVPTPSGRTVSISEAPEDVAATYRRQVMEKTKETLLKPYKKMCER<br>KKVAVELQVLESNSVEVAITREVNQHLISRLVIGGSSHVGLDGNRDVTAKISAYVSELCT<br>VYVVSKGVYILSKNKSSSDGEINETIRDSGSERTDTSSYSSSSGHISAALKSKSLVLSNK<br>RLQHLPTIARGVSVRMETSSVDSYETKSMFSDQVSKRSSPETSRTVTWNPPRRYMSSNEN<br>VTQGDDYFTDNQDTLHEIRKLKDELRQAQEIYAMAQVETLDASRKLNELEFEELKLKENG<br>TKGLEEKETQKFDQMRRETREVAQKREAREKEDKLKESLEDRLFQVNKSQPIPWFLRFRI<br>AWEVASALIFLHKSKPTPIIHRDLKPANILLDQNFVSKVGDVGISTMLQVDPLLTQFTTY<br>KKTSPVGTLCYVDPEYQRSGMLSFKSDVYAFGMIILQLLTALPAIALTYKVETAMEKNVE<br>FIQILDKKAGDWPMEETRKLAALALSCTEIRVKDRPDLETQILPALESFKNAAEKVRNLI<br>SSASKQPPRHFLCPLLKDVMSDPCVAADGYTYDRRAIEEWMEDHCTSPVTNLPLQNIKLL<br>PNHSVCAAIVEWRHTNQ*
BoPUB42	MSTSKPQRSPSEIEDIILRKILYVTLADSATDPRVVYLEMTAAEILSEGKPLLLSRDLME<br>RVLIDRLSGNNNFPSSSAAEPPFPYLIGCYRRAYDESKKIQSMKDKNLRSEMETVTRDAR<br>RLAVSYCRIHLANPELFGGSDDARKAKKRRNVSPLLPLIFSEVGSGSLDMFGGGGGSSGG<br>VQSPPGFLDEFFKDSDFDNLDLILKELYEDLRSSVINVSVLGDFQPPLRALKYLVSLPVG<br>AKSLVSHEVWVVPRGAYMNGRAMELTSILGPFFHISALPDNTLFKSQPDVGQQCFSGASER<br>RPADLLSSFSTIKNFMNILYSGLHDVLMILLKSTDTRECVLQFLSEVINANASRAHIQVD<br>PVSCASSGMFCNLSAVMLRLCEPFLDPHFTKRDKIDPKYAFAGHRLKLSDLTALHASSEE<br>VSEWIDKGNTAKANDAGNGNESRLLQSKEATSSSSSSQQNAKSTTKYTFICECFFMTARV<br>LNLGLLKALSDFKHLSQDISRGEDNLATLKAMRDQAPSPQLELDITRMEKELELYSQEKL<br>CHEAQILRDGDFIQRAISFYRLVIVWLVGLVGGFKMPLPSACPMEFSCMPEHFVEDAMEL<br>LIFASRIPKALDGVLLDDFMNFIIMFMASPEYIRNPYLRAKMVEVLNCWMPRSSGSSATS<br>TLFEGHQLSLEYLVRNLLKLYVDIEFTGSHTQFYDKFNIRHNIAELLEYLWQVPSHRNAW<br>RRIAKEEEKGVYLNFLNFLVNDSIFLLDESLNKILEIKQIEAEMSNTAEWEQRPAQERQD<br>RTRLFHSQENIVRIDMKLANEDVTMLAFTSEEITAPFLLPEMVERVANMLNYFLLQLVGP<br>QRKSLSLKDPEKYEFRPRQLLKQIVRIYVNLARGDSENIFPSAISSDGRSYNEQLFDAGA<br>DVLRRIGEDGRIIQEFMELGTKAKAAASEAMDAEAALGEIPDEFLDPIQCTLMRDPVILP<br>SSKTTVDRAIIQRHLLSDNHDPFNRAHLTSDMLIPDIELKARIDEFVRTHQSKKRSSGED<br>SSNKERIQTTSSDMLID*
BoPUB43	MGTETYITVPSFFKCPISLDVMRSPVSLCTGVTYDRASIQRWLDGGNNTCPATMQVLRTK<br>EFIPNLTLQRLIKAWSDSVGRYAAASPPPDPTLKEVNESLRRLSLEKDDEIRLEILVRFV<br>KDSDANRAFLSARKDLVPMLVDIIAGGTRTTTRIKLVLLAIKILDSITNGGSEGDRKRLS<br>NLMLTNGGGDCLTAILLAIQRGKLESKIESVRVLEVVSSFDAKSKLLIAERDEILTEVIK<br>SISTESDPTLIEASLSFLITIAKSKRVRSKLIAAKTITKIKDILLTEETPSVAVTEKSLK<br>LLETLTSKREGRSEICRGDGGRCVEGVVRKLLKVSTTATKHAVTILWCLCYVFGEDKRVK<br>ETVERSNGVTKLLVVIQSNCSPMVRQMAKDVIKVLKVKPSASDLVAYETKTTHIMPF*
BoPUB44	MPRSLEPLDLGIQIPYHFRCPISLELMRDPVTVCTGQTYDRTSIESWVSTGNNTTCPVTR<br>APLTDFTLIPNHTLRRLIQEWCVANRSNGVERIPTPKQPADPTSVRALLSQASATSGTHV<br>SARSRAAALRRLRGFARDSEKNRVLIAAHNAKEILIRILFSDDIDSSELVSESLALLVMF<br>PMTEHDKCVSIISDPGRVEFLTRLLFDSSVETRVNAAALIEMAVTGSKETVSNSESIFEG<br>VLDLLRNPASSYPRRALKIGIKALFALCLSKNTRHVAVSAGAPEILIDRLAAGLDRCDTE<br>RALATVEILCRSPEGCAAFGEHALTVPVLVKTILRVSDRATEYAAGALLALCTAEDRWRD<br>EAAAAGVVVQLLLMVQSECTERAKRKAQKLLKLLRDSWPDYSFANSDDFACSEVVPF*
BoPUB45	MAKTELAKILIESVKEIASISDHRPPMKIHCAHLSRRLKLLLPMLEEIRDCKNSLPEESM<br>MKALLSLRESLLHAKDLLIYISQVSKIYLVLERDQVMVRFQKVTALLEQALSDIPYQSLE<br>ISDELQEQVELVLVQLRRSIGKGRGEKYDDELYKDLLSLYDGATDSEILRRVAEKLQLMT<br>VTDLTEESLALLDMLSTSGGDDPGESFEKMMMLLKNIKDFVQTYNPNLDDVPLRSKPSLS<br>KAQDEDEKNVPEDFRCPISFKLMSDPVIVSSGQTYDRDCIKKWLEEGNSTCPKTQATLSS<br>DTVTPNYALRSLIAQWCESNGIEPPKRPNNPQPSSKASSSSSSSSSSFSPPSDEQLKIEA<br>LLCKLTSQRPEEQRSAAGEIRLLAKQNNHNRVVLAASGAIPLLVSLLMNFDDPRTQEHAV<br>TSVLNLSIFQENKGRIISTFGAVPGIVQVLKKGSMEARENAAATLFSLSVIDENKVTIGA<br>AGAIPPLVNLLSEGSQRGKKDAGTALFNLCIFQGNKGKAVRAGLVPVLMRLLTEPEGRMV<br>DEALAILAILSSHPEGKVVVGAADAVPFMVDFIRSGSPRNKENAAAVLVQLCSWDPQYLI<br>EAEKLGLMGCLIEMAENGTDRGKRKAAQLLNCFSRFNEQQKQSGLGVEGQVSLI*

BoPUB46	MEESTAESTAANADTLKVELKKLLIEILSNGGGEIEDRGESDASSGVLKAIDEAIRLLNR
	LREVESKKPESDIPSSSSSQSPKVEVPKEFKCILSNAIMIDPVTIASGNTYEKSYITELL
	KHAPKCPKTNEVLSHSLCTPNHLLDELITKWCIDNGYDRLKPPHEVVTELSRDGIESLLQ
	RISSPSSDEDQNEAAKEIRCQTNKFPSVRNRFVALHPGAITTLIRPLSNFITKPELRENI
	ITALFNISIVMENKEAIAQTDNVVGELRFSLSCGTMESRRNSASTFLSLLAIDSNKEIIG
	SKLMLIGLGSLIGEGETVTSLVAGSVVYQLCQDLKYREMATSAGVVPALMGKIEAGSYAA
	EFLIVLALLTTYERGVKEMEKRPEFIKHLFSILRKRSCLMSCENAVLIVINMVVNLGRVAI
	VNEEENKHATFAMLRKEESSVSLATNADMVLQWPKVYGTGKAPAIESRRRSFKVKSKLLA
	PDQFALINPSFDLDPLQDPPQTTAPSGTGNGKIRYRSPSSPELMESGTAAVSSSGHHSPS
	HSSESHQGLLSVDGGKTTTAKRGIGRHESITDKIQRHRGILLLISVAILLIGLVLLLMPG
	RSTSDAVVEEYTVLNRKGGPNSRPPKNYAVIFDAGSSGSRVHVYCFDRNLDLLPLGNELE
	LFVQLKPGLSAYPTDPRQAANSLVSLLDKAEASVPRELRPKTPVRVGATAGLRTLGHEAS
	ENILQAVKELLRDRSMLKTEANAVTVLDGTQEGAYQWVTINYLLRNLGKPYSDTVGVVDL
	GGGSVQMAYAISEEDAATAPKPLEGEDSYVREMYLKGRKYFLYVHSYLHYGLLAARAEIL
	KVSEDSNNPCIVAGYDGTYKYGGDGFKAAAVQSGASLNECRRLTVNALKVNDTLCTHMKC
	TFGGVWNGGRGGGQKNMFVASFFFDRAAEAGFVDPKQPVATVRPIDFEKAAKKACSMKMD
	EGKSKFPRVEEDNFPYLCMDLVYQYTLLVDGFVKKVKYGEHAVEAAWPLGSAIEAVSSP*
BoPUB47	MEELTAANADTLRMELKKTMTEILDVGGVSEDRSETDGALVAVDEAVRILNRLREVESKM
	PDSATSSSSPASVLEVPKEFKCMLSRAIMSEPVVIASGQTYEKRYIQQWLMYKVTCPKTK
	EVLSHRLWSPNHVIAELITQWCQANKCDLPKPSDAPIGLFPDDIDLLLHRISSPSSVEDQ
	TGAANELRRQTKRFADVTAFFVAEIPDSITRLLTPLSALGEAIDSNPGLQKNIITALLYI
	SSLEENKTAVAQNPIVIPLLTKSLKQGTAKTRRNSANALWELSKLDSNKIHIGKSETLEA
	LVLVIKEDHFTAAIGAAYAVFQLCCLPENRGKVVSEGLVPALINRIKERSYVYIYFALLS
	LMTIQKGVIEEMEDLGFVDDMCSILRNTSCSVTEENGVSIVLLRMCGLNTGDKDRERTRL
	KLIKEEEDKYSTFTKFATQGSQCAVAEAEAVLQWIKRSVTGEEP*
BoPUB48	MEESTAESTAENADTLRMELKKTMTEILGDGGVSEDRGEADGSSRVLKAIDEAVRILNRL
	REVESKMAESADTSSSSPAASVLQVPKEFKCMLSRAIMSEPVVIASGQTYEKRYIQQWLM
	YKQTCPKTKEVLSHRLWSPNHLVDELIAQWCQANKYDRPEASDAPIGLFSDDIDLLLERI
	SSPSSVEDQTKAANELRRQTKRFANVTAFFVAEIPDSITRLLTPLPALGEVIDTYPGLQK
	DIITALLYISSLEENKTAVAQHPLVIPLLTKSLKKGTAKTRRNSAEALWLLSKLDSNKII
	IGNSETLKALVHVIKEDHFTAAISASYAVFHLCCLPENRGKVVSEGLIPALINRIKERSY
	VYIYFALLALMTAQNGVIEEIENLGFVDGMFSILRNTSCSVTGENGTLIFLRMCGLNTGN
	RDRERTRLKLIKEEEEKYSTFSKFAKQGSERAVMEAKAILQWIKRSGTGEEP*
BoPUB49	MIPTQNRRILTFTAVHPCESVSITTLIDSLIKIAGEILSFKSKHFSTNKRSVKETLRHVL
	NLLIVFQEIRVGLIPADRSFPRSAILSLSELHIIFQKLVILLEECTRKGGKLYMLTNSDQ
	ISAHFRVLTRSISTCLDTFPVGSIELPEEVKELIYLLIRQTRKYEARPDRDDKRALDSVY
	WIFNLFENRINPNRDEVVRVLDHVGVRKWRDCVKEIGFLGEEITVEGKDKNEIELLSSLM
	GFICYCRCVILGGIDEDEDKGEEEEDDLVIRGLNVDDLRCPISLEIMNDPVVLETGHTYD
	RSSIAKWFSAGNITCPKTGKNLESTVLVGNVSVKQVIQSYFKKEIDQKSMKKKKKKTSIP
	ESLAAEEAGKLISDFLAGELINGGPKEMVKALVEIRILTKTTSFNRSRLVEAGVVESLMK
	LLRSGDPTIQENAMAAILNLSKDISGKVRIGGSGGGLEMIVEVLNEGARRESRQHAAAAI
	FYLSSLGDYSRSIGEIPDSIPGLLRIVKGCDYGDSPKRNALIAIRSLLIQHSDNHWRVLA
	AGAVSVLLDLVRSGEIGDGVKADSIAVLAKIAEYPDGMISVLRRGGLKLAVKVLGSSEVS
	PVTKQHCVVLLLNLCVNGGSDVVGALAKDPSVMGSLYTALSNGECGGGRKASALIKMIHE
	FQERKTETGLERERFIHAW*
BoPUB50	MIHTKTGSNRRILTFPAVQPCKSISVTTLLDSLIQLAGDILTFKQKHFSTNKRSFQKTIR
	QIQNLAIVLEEIRIRVGSRRRYFPGVSSLSEIHVIFQKLKFLLEDCTRDGGRLCMLMSSD
	QVSDHLRVLTLSISTSLSAFPVSFVDLPIEVNEVIGLVVQQAREHVVRPDSDDKKVIASV
	NGVLALFENRVIPDPDEINRILDRVGVRTWGDCLKEINFLGEEIDAERLENKKKNNNSNA
	RVELLSSLMGFICYCRCVILNKRDHHHHHHDESELRVHEDLIRGIKVEDLLCPISLEIM
	TDPVVIETGHTYDRSSITKWFGSGNITCPKTGKILTSTELVDNVSVSHVIQKHCRANGVV
	LEINGRKRKSHGDVAPDSLAAKGAGRLIARFLTSELLTNGDEEMVYRALREIRVLTKTGS
	FNRSCLVEAGCVSPLLNLLTSEDSRIQENAMAGLLNLSKHVAGKGEIAGEGLGIIVEILN
	EGAKTETRLYAASALFYLSSVEDYSLLIGEHPDAIPGLMRIVTGDEYGDSAKRNALLAVM
	GLLMQSENHWRVLAAGAVPALLDLLRDEETGGELTADCLATLAKLAEYPDGTIGVIRRGG
	LKLAVKILSSSEVSPGVKQHCVGLVLNLCLNAGNDVVGLVLVKDSVVMGSLYTVISNGEYG
	GSKKASALIRMIHEYQERKTGSVEPSFQRGRFIHAW*

BoPUB51	MDEEIEIPPFFLCPISLEIMKDPVIVSTGITYDRDSIEKWLFSGKKNSCPVTKQDITDAD<br>LTPNHTLRRLIQSWCTLNASYGVERFPTPRPPICKSEIEKLIKDSASSHQNQVKCLKRLR<br>QIVSENKTNKRCLEAAGVPEFLATIVSNNESQNVFDSSMSLTDEALSLLYHLETSETVLK<br>NLLNKKKGRDIVSSLINIMQRGMYESRAYATLLLKNILEVAEPMHIMNLKPLVFTEVVQI<br>LEDRISHKATKAALHILVNICPWGRNRHKAVEAGAIYVMIELLMDESFSSDRRGQEMAMV<br>VLDLLCQCAEGRAEFLNHGAAIAVVCKKILRISQTASDRAVRVLLSVGRFCATPALLHEM<br>LQLGVVSKLCLVLQVNCGSKTKEKAKELLKLHARVWKDSPCLPRNMILAYPS*
BoPUB52	MATDSAMFASSRRRQSPSLEAFLSPVDLSDVPLLRTLSSTSSEIVSCFSNARFSFQRRNT<br>RSLIRKVQVIAVLLQHLAPESSLDPTAVLCFKELYLLLHHSKFLLRYCAHSSKLWLLLQS<br>PSLSSFFHDLSKDYSTLLDVLLPAESLCLNDDVREQVQLLHMQHYVDDNNDETLRNRLYS<br>FLDEFENGSVPNSEELSFFFFEKLGIKDPTSYRDEIEFLEEKIKTYGCDLEPTRSVINGF<br>VDITRYVMFLLFKIEDSNEIKKQRKGLISEEIENTFTTLPKDFICSISLNIMNDPVVIVS<br>TGQTYDRSSIARWIHQEGRSTCPKTGQKLVDLSFVPNLALRHLTTLWCQVNGLSHDSPPP<br>KESLPKVFQTRASTEANKAAISILVRNLAHGSELAAGEIRVLTRTVTETRTLIVEAGAIP<br>YLRSLLKSENAVAQENAVASIFNLSIDEANRSLIMEEHDCLEPIMSVLVSGLTMRAKEIA<br>TAALYTLSSVHDYKKTIANADGCIESLALVLRNGTVRGKKDAVYALHSLWLHPDNCSLVV<br>KRGGVSALVGALGEESVAEKVACVLGVMATESLGAESIGREETVVTGLMELMRCGRPLGK<br>EKAIATLLQLCTLGGAVVTEKVVKTPALAVLTRKLLLTGTDRAKRKAVSLSKVCKGCDQK<br>TQR*
BoPUB53	MKSNVFDGAPPDYSSISVAVKGSIGDTVGGAASRRAVRWAVDNLLPHIDRLVLVHVMPTV<br>TTIPSPSGSKIPVEDLEESVVSMYKQDLRKEFEEVFVPFNKICGSIKAETLLLENDDPAK<br>ALLKYVSDSEVECLVIGSCSPSFLTRQERRFGKKKGQEMPLMVLGEAPETCEVYVIAKDR<br>VLTKSTNQLSPVKLSSESLYSFRTPKRGEAHTDPFNRTCSDKTGLAASYMSPSPARNQIR<br>RPVSLPPSHQASRVFSPAQASTGIRLGHDERVRSILGHNIVSSSNMQLNPGANMNTPKWQ<br>SNVMYEVEQLRKQVQTTLCMYKQACEELVHKQTQVQSLSYECIKDTKRVISALEKEEMLR<br>KEAEEEKQKHLKAVKEIEEAKTVLAKEFCDRKLAELNALQQALKKQQVMDQLLLSDSRYR<br>KYTKEEIVAATDNFSLSKIIGEGGYGKVYKCSLDHTPVALKVRRPDTIEKKQEFLREDAV<br>KRGCFGDMLDGSVREWPLAEAEELARIAIQCSQLKCRDRPDLDTQVLPALKRILESAKSK<br>LKTEQAKARPPSHYYCPILKEIMEDPQIAADGFTYEGKAIKAWIQNNQNVSPVTKNRLRH<br>CDLAPNHTLKSAIQEWRSRSGLDLSTTLGSS*
BoPUB54	MDEEIEIPPFFLCPISLEIMKDPVIVSTGITYDRDSIEKWLFSGKKNSCPVTKQDITDAD<br>LTPNHTLRRLIQSWCTLNASYGVEKIPTPRSPICKSEIEKLIKDSASSHKNQVMCLKRLR<br>QIVSENATNKRCLEVAGVQEFLATIISNNGFDSSMSLSDEALSLLYHLESSETVLKNLLN<br>NKKGGNIVKSLTKIMQRGIYESRAYATLLLKNILEVADPMQIMTLKPSVFTEVVQILDDR<br>ISHKATKAAMHVLVTTCPWGRNRHKAVEAGAISVIIELLLDESFSSDRRGPEMAMVVLDM<br>LCHCAEGRAEFSNHGAAIAVVCKKILRVSQTASDRAVRVLLSVGRFCATPALLQEMLQLG<br>VVEKMFLVLQVSCGSKTKEKAKELLKLHARVWKDSPCLPRNMILAYPS*
BoPUB55	MGETLHNDVIYVAVNQDVRESKSTLLWTLKTLPVKKLCLVHVHIPFSLNSSSCGLDESEI<br>NAIQESELKSSYDSLHKYRDLCTNKEVDEEDVDISLISGYGVGEGIVELIYQNNIKKLVM<br>GAAADPHYSRGMSITSRKAEYVSQHAPHCCKMWFICKGKLIQKKEGSFGIGNPSDSFSGF<br>DGFAQKPSRGRGIDSDDQPNSRKEDSGRNQTPKEVRKEDKPKSNDSETDSPPENFICPIS<br>MEIMRDPHVAADGFTYEEKEIRTWLNGGNDKSPITGARLAHRHLTPNYTLRSLIKDWLHL<br>HPNYKH*

BoPUB56	MDMAEEAWIDDPVYVAVNKDAGESRSTLTWALRHLQFKKLFLLHIHLPISMNPTSSGLEQ<br>SEIDAIQVSELTSTYESLLKYRDICLHEGVNEQDVEIFCDVGNNVEEGIVNLIYENNIKK<br>LIIGAAADSRYSEGMVNITSRKAKYVSSHAPHCCKIWLVCNGNLIQTREGRFDLEGSSHS<br>SSESLASLHDLDSALIPFEDVVRAEPDSESHALSSPEDQSARGSAATYYEEQRRRLEIEE<br>LKRELEQHDKMNREREEALSSSFGVTQMLYSEEVRRRREAEEDLNRARAEIKDMKRVQKE<br>LEEQLYIDCPRRLDMVEKERDEAIKKTEELLGNLHLEKGESSSHSASSSSQWSVSNEPPP<br>YFICPISKEIMQNPHVAADGYTYEADEFKNWLVHGGEKSPMTNLKLVNHNLTPNLALRSA<br>INEWLQQHPYFFDLP*
BoPUB57	MAELMAMGNDVVHVAVKNDVKESRSTLVWALRNLGAKKVCILHVYQPKTASPAARKLEEL<br>EAIMYETLHDYFDICQQEGVNEDDIYISCIEMNDVKQGILELIHESKIKKLVMGAASDRD<br>YSEKMFDLKSRKAKYVYQHAPSSCEVTFMCNGHLIYTKEANLEDRVDETEAEAGQWKPKL<br>YSSASPKCSAELVSAIVAYIDTRRDRDMLEPTASEDQSESDRNDQLYRQLKQALIEVEES<br>KREAYEECEVMREPRVAADGFTYEAEALKEWLDNGHETSPMTNLKLTHNNLVPNHALRSA<br>IQEWLQRNS*
BoPUB58	MEEEKGAVAQSLIDVVTEIASVSDYRITVKKLCSNLARRLKLLVPMFEEIRESNDPISED<br>TLGTLVSLKEALSSAKDHLKFCSGGSKIYLVMEREQVTSKLLEVSVQLEQSLSKIPYEDL<br>DISDEVKEQVELVLSQFRRAKGRVDASDDELYQDLHSLCIKSSDVDDHQPALQRVANKLQ<br>LMEIPDLAQESVALHEMVALSGGENNEEMAMVLKLIKDFVQMETDNSEDQKVGVSNGQTS<br>TAACQKIPPVIPDDFRCPISLEMMRDPVIVSTGQTYERTCIEKWIEAGHSTCPKTQQALT<br>STTLTPNYVLRSLIAQWCEANDIEPPKPPSSFRPRKVSSFSSPAEANKIEDLMWRLAYGN<br>PEDQRSAAGEIRLLAKRNADNRVAIAEAGAIPLLVGLLATPDSRIQEHSVTALLNLSICE<br>NNKGAIVSAGAIPGIVQVLKKGSMEARENAAATLFSLSVIDENKVTIGALGAIPPLVVLL<br>NEGTHRGKKDAATALFNLCIYQGNKGKAIRAGVIPTLTRLLTEPGSGMVDEALAILAILA<br>SHPEGKSIIGSLDAVPSLVEFIRTGSPRNRENAAAVLVHLSSTDPQHLVEAQKLGLMGPL<br>IDLAGNGTDRGKRKAAQLLERISRLAEQQKETAAHVQTKEEAEPTDSASTTEAADT*
BoPUB59	MDVSELEENLFAVSDAKLHRDMCKELSAVYCKVLSIFPSLEEARPRSKSGIQALCSLHIA<br>LEKAKNILQHCSESSKLYLAITGDAVLLKFEKAKSALIDSLRRVEDIVPSSIGSQILEVV<br>GELEHTKFLLDPSEKEVGDRIIALEIFHHAATRLSITSSRSALAERRALKKLIDRTRAEE<br>DKPSPTGNEDRAHHAFGRQLSKFGSINFKKSGQAPTPPEELRCPISLQLMCDPVIIASGQ<br>TYERVCIEKWFGDGHNSCPKTQQQLPHLSLTLNYCVKGLIASWCEQNGISVPAEPPESLD<br>LNYWRLALSDTESANSKSVDSKGSSTLKAPETVPLEERSTIDKEDVTVADEETSEINVLE<br>KYQDILAMLEKEDLAKKCKVVENVRLVLKDDEEARILMGANGFVEAFSRFLGSAVDENNA<br>AAQETGAMALFNLAVNNNRNKELMLNSGVIPVLERMISCSHSQGPATALYLNLSCLEKAK<br>PVIGSSQAVPFFVKLLVQVENTETQCQLDALHALYNLSTHSPNIPTLLSSNIIKTLQLLV<br>STGDHLWIEKSLAVLLNLASSQEGKEEMISSQGMINTLATVLDTGDTIEQEQAVACLVIL<br>CTGSERCIQMVLQEGVIPSLVSISVNGSPRRRDKSQKLLMMFREQRQREQPSLNKEEAPR<br>KSVSAPLPMSVSGQASSPESEGKPLFKSISRRKTLTRPLSFLWKKSYSLHH*
BoPUB60	MATAAVFSSLRRRKSPSLEAFLAPVDLSGTALIQTLASISTEIISCFAAIRFSFQRRNAR<br>SLIRKIEIFLVLFEFLAESRWGSSSSSSTALLCLKELYLLLYRSKILLDYCAHSSKLWLI<br>LQTPSISGYFHDLNQEISTLLDVLPINGLDLTDDIREQIELLRSQSRRSRLYIDNHDESL<br>RQTLYSFLDGFENAEIPNASSLKTFFPEKLSIKDSESCTAEIEFLEDQIANHDGDLEPTG<br>SVINAFVAIARYCRFLLFGFEEDGSEWRRKPSKEEIGRGETLITVPKDFVCAISLELMLD<br>PVIISTGQTYDRSSIARWIEEGHCTCPKTGQMLTDSRIVPNRALKNLIVQWCTASGVSCE<br>SEFITDSPNEGFVSALPTKAAVEANRATVSILVKYLAEGSEAAQTVAAREIRLLAKTGRE<br>NRECIAEAGAIPHLRRLLKSENAVAVENSVTAMLNLSIYEKNKSRIMEEDGCLECIVSVL<br>VSGLTVEAQENAAATLFSLSAVHDYKKRIASVDQCVEALASLLQNGTPRGKKDAVTALYN<br>LSTHPDNCSRMVEGGGVSSLVGTLKSEGVAEEAAGALALLMRQPLGAEAIGKEEAAVTGL<br>MGMMRCGTPRGKENAVAALLELCRGGGAAVAERVLRAPAIAGLLQTLLFTGTKRARRKAA<br>SLARVFQRRENAAMRSGGYGFVGNGNGTRDGGSFTTDVSVPMSISISVPVL*

BoPUB61	MSGKGLSPASFSAPPMTVQDADPLRFRVGEQDPKTREFAAFIGDQHRYFAAAAAAAAAN
	PHPQPHIEFRQSFYSESGNPNDSGGSDGEEVVEEEEEEEEDDDDDVDGNEGDGMNKDVGE
	DGGRAVVGIEQDNAAYYSQQFKAMEASFVSRHEESSSVAAENGCDVSGRRDASSSSLSSS
	IESLRTILSDPITGALMSDAMILPCGHTFGAGGIEQVKQMKACCTCSQPVSEDSITPNLT
	LRVAVQAFCREEKSQSNHSSKRKREGFDQERRSFGDTAYTNRSRNRTNHFPFAVADRVII
	KGNKRTPPRFVGREAVVTTQCLNGWYVVKTLDNAESVKLQYRSLAKATEDPSTKATPSKM
	VSNWL*
BoPUB62	MDPVPVRCLLNSISRYLHLVACQTIRYKPIQTCIGNVVHLLKLLKPFLDDVVDCKVPPDD
	CLNSACEDLDSVVNQAREFLEDWSPNVQGVEVNLLQRCMQEIECFKQERTLSEHMKDTVP
	LDTDDLDSIIQMMGLISNQDLLKESIAVEKARIRSQTSKSKEKIEQIDQLIDLVSCIREH
	MLKTEFLEVAKGVSIPPYFRCPLSTELMLDPVIVASGQTFDRTSIKKWLDNGLDVCPRTR
	QVLTHQELIPNYTVKAMIARWLEENSINLPANSDGGGDASSMANNRGSNDFNRTESFRFS
	LRSSSFTSRSSLETGNGFEKVKINVPASLCGESQCKDVENFELSSSGQSYTHSRSESVCS
	VVSSVDYVPSVTNETQSIQENHQSCSEMSPKKHSESSSNVDHVYSECSSHTMKLVQDLKD
	GSSKEKTAAAAEIRHLTINNVENRVHIGRCGAITPLLSLLYSEEVLTQEHAVTALLNLSI
	SEVNKAMIAEAGAIEPLVHVLNTGNDRAKENSAATLFSLSVLQVNRERIGQCNAAIQALV
	NLLGKGTFRGKKDAASALFNLSITHENKARIVQAKAVKHLVELLDPELEMVDKAVALLAN
	LSAVGEGRQAIVREGGIPLLVETVDSGSQRGKENAASVLLQLCLNSPKFCTLVLQEGAIP
	PLVALSQSGTQRAKEKAQQLLSHFRNQRDARMKKGRS*
BoPUB63	MEIDPVTNGNTDTVMTEPSPAIAPSRPVSSSRSSQLTESIKLEHQLLRVPFEHYKKTIRA
	NHRSLEKEVSSVVSSVGDLADNDWSKDVAVSRLTSLVSRLQGLKRKLEEGSNVENLQAQR
	CRARIDHLDSADVENITEWNNTKLKRILVDYMLRMSYFETASKLSESSNISDLVDIDIFR
	EAKKVIDALKRREVASALTWCADNKTRLKKSKSKLEFQLRLQEFIELVRADSYKQAILYA
	RKHLTPWGATHMNELQHVLATLAFKSTTECPKYKALFEPQQWDVLVHQFKQEFCKLYGMT
	MEPLLNIYLQAGLTALKTPYSFEEGCTKEDPLSQESFRKLALPLPYSKQEHSKLVCYISK
	ELMDTENPPQVLPNGYVYSTKALKEMADKNKGEIKCPRTGFVCNHTDLVKAYIS*
BoPUB64	MGCAASLPGKFSPFHRNSGALNGLTSSENAAPADAKNLRVKLVLLGDSGVGKSCIVLQFV
	RGQFDATSKVTVGASFLSQTITLQDSTTVKFEIWDTAGQERYSALAPLYYRGAGVAVIVY
	DITSPESFKKAQYWVKELQKHGSPDIVMALVGNKADLHEKREVPSEDGMELAEKNGMFFI
	ETSAKTADNINELFETLFFLFVLATCKNSQSCQKTEKLERGYLVLLNHHHDWSVWIIPSS
	AKSINLSPGVLPPFNHFLLLRLELSGMGSTNEELMSRLVDSVKQISKFSNSKGFFGRIQG
	DLVRRITLLSPFFEELIDIGAELNEEQQLTGFEVMMIALDSSLQLFRSVNGGSKLLQIVH
	KGSVMQKFHDVTVEIEAALTQIPYDHFEVSEEVIEQVKLLHSQFKRATERQEDYDLQLSH
	DLAVAESVVDPDPETLKRLSQELQLSTIDELKKESHAIHEYFLSYEGDPDDCFQRMSSLL
	KKLVDCASTESSEPDASTGSKVISRHRSPVIPEYFRCPISLELMKDPVIVSTGQTYERSS
	IQKWLDAGHKTCPKSQETLLHSGLTPNYVLKSLIALWCESNGIEFPQNQGSSYRTTRPGG
	GSSSSDCDRAFVLALLEKLANGSTEQQRAAAGELRLLAKRNADNRVCIAEAGAIPLLVEL
	LSSPDPRTQEHSVTALLNLSINEGNKGAIVDAGAITDIVEVLKNGSMEARENAAATLFSL
	SVVDENKVAIGAAGAIQALISLLEEGTRRGKKDAATAIFNLCIYQGNKSRAIKGGIVDPL
	TRLLKDAGGGMVDEALAILAILSTNQEGKAAIAEADTIPVLVEIIRTGSPRNRENAAAIL
	WYLCIGNMERLNVAREVGADVALKELTENGTDRAKRKAASLLEIIQQLEGVVISTVP*
BoPUB65	MGTSALVDSISSFLNLSSSRHIDLDPFEKYYKRVEELLIVLKPVADAAATNSDLVSDERI
	GKSFDDLTQDVDHSIDLFRSWHAFSSKVYFVLQIESLIPKMRETIVDTFHFLMSFESNLP
	DDLTPPSLEQCLEKVKQLSYEEISSVIDGALRDQRDGAGPTPDVLVTIGENTGLRSNQEI
	LIEAVALERQKEIAEQSENNAEVEFLDQLIVIVNRMHERLLLIKQTQTSTVAILADFFCP
	LSLEVMTDPVIVSSGQTYEKAFIKRWIDLGLKVCPKTRQTLTHTTLIPNYTVKALIANWC
	ETNDVKLPDPNKSTSLNELSPLLSCTESAPSVSTHRVSNKSNDWDASTGETVKPPSSSNL
	HPSPSPSRSASALDTSSLHDYEVRSNDSREMKTDAPGRSSVSSTTRGSVENGQTSENHHH
	LRSPSAASSVSNEDSPRADANENSEESPHATPYSSDASGEIRSGPLAAATTSAAPPRRDL
	SDFSPKFMDRRSRGQFWRRPSERLGSRIVSAPSSETRRDLIEVENQVKKLVEELKSSSLD
	VQRQATAEIRLLAKHNMDNRIVIGNSGAIVLLVELLHSTDSATQENAVTALLNLSINDNN
	KSLIAQAGAIEPLIHVLQNGSSEAKENAAATLFSLSVIEELKIKIGQSGAIAPLVDLLGN
	GTPRGKKDAATALFNLSIHQENKGTIVQSGAVRYLIDLMDPAAGMVDKAVAVLANLATIP
	EGRNAIGQEGGIGLLVEVVELGSARGKENAAAALLQLSTNSGRFCNMVLQEGAVPPLVAL
	SQSGTPRAREKAQALLSYFRNQRHGNAGRG*

Table S3. Conserved domains in the BoPUB protein sequences.

Name	Accession[a]	Molecular function[b]	Biological function[c]
U-box	PF04564.15	Ubiquitin-protein transferase activity (GO:0004842)	Protein ubiquitination (GO:0016567)
Arm	PF00514.23	Protein binding (GO:0005515)	——
Arm_2	PF04826.13	——	——
Arf	PF00025.23	GTP binding (GO:0005525)<br>GTPase activity (GO:0003924)	——
Prp19	PF08606.13	——	——
WD40	PF00400.32	Protein binding (GO:0005515)	——
Ufd2P_core	PF10408.9	Ubiquitin-ubiquitin ligase activity (GO:0034450)	Protein ubiquitination (GO:0016567)
TPR_8	PF13181.8	Protein binding (GO:0005515)	——
Pkinase	PF00069.25	Protein kinase activity (GO:0004672)	Protein phosphorylation (GO:0006468)
PK_Tyr_Ser-Thr	PF07714.19	Protein kinase activity (GO:0004672)	Protein phosphorylation (GO:0006468)
Usp	PF00582.28	——	——
CLTH	PF10607.11	——	——
GDA1_CD39	PF01150.19	hydrolase activity (GO:0016787)	——

[a]Accession number from the Pfam database.

[b]Molecular function of conserved domains from the InterPro database.

[c]Biological function of conserved domains from the InterPro database.

Table S4. Ka/Ks analysis of duplicate *BoPUB* gene pairs.

Duplicate gene 1	Duplicate gene 2	Ka	Ks	Ka/Ks	Gene duplication type	Evolution type
BoPUB1	BoPUB34	0.039293141	0.40990778	0.095858489	Segmental duplication	Purifing selection
BoPUB5	BoPUB14	0.106800124	0.363841159	0.293535026	Segmental duplication	Purifing selection
BoPUB9	BoPUB6	0.062123453	0.531965695	0.116780938	Segmental duplication	Purifing selection
BoPUB11	BoPUB60	0.0768889	0.516289517	0.148925937	Segmental duplication	Purifing selection
BoPUB12	BoPUB34	0.043030074	0.33961439	0.12670274	Segmental duplication	Purifing selection
BoPUB16	BoPUB34	0.106618211	0.975271982	0.109321516	Segmental duplication	Purifing selection
BoPUB26	BoPUB42	0.031392483	0.258291075	0.12153917	Segmental duplication	Purifing selection
BoPUB43	BoPUB30	0.108978754	0.447109283	0.243740754	Segmental duplication	Purifing selection
BoPUB51	BoPUB34	0.11817211	1.097901566	0.107634522	Segmental duplication	Purifing selection
BoPUB54	BoPUB51	0.052214102	0.339894367	0.153618614	Segmental duplication	Purifing selection
BoPUB46	BoPUB47	0.44074428	0.605545475	0.727846709	Tandem duplication	Purifing selection
BoPUB55	BoPUB56	0.384430852	1.265418538	0.303797392	Tandem duplication	Purifing selection
BoPUB56	BoPUB57	0.480420197	2.304581439	0.208463103	Tandem duplication	Purifing selection

Duplicate *BoPUB* gene pairs were identified by the MCscanX package, and the nonsynonymous substitution rate (Ka), synonymous substitution rate (Ks), and Ka/Ks ratio of each duplicate gene pair were calculated by the KaKs_Calculator 2.0 software.

Table S5. Prediction of interacting BoPUB protein pairs.

Interaction protein 1	Interaction protein 2	Coexpression	Experimentally determined interaction	Database annotated	Automated textmining	Combined score
BoPUB7	BoPUB1	0	0	0	0.461	0.461
BoPUB7	BoPUB6	0	0	0	0.529	0.529
BoPUB7	BoPUB9	0	0	0	0.529	0.529
BoPUB7	BoPUB11	0.043	0	0	0.516	0.517
BoPUB7	BoPUB12	0	0	0	0.461	0.461
BoPUB7	BoPUB16	0	0	0	0.461	0.461
BoPUB7	BoPUB19	0	0	0	0.51	0.51
BoPUB7	BoPUB23	0.045	0	0	0.531	0.532
BoPUB7	BoPUB26	0.042	0.814	0	0.71	0.943
BoPUB7	BoPUB30	0	0	0	0.51	0.51
BoPUB7	BoPUB34	0	0	0	0.461	0.461
BoPUB7	BoPUB36	0.045	0.086	0	0.506	0.531
BoPUB7	BoPUB42	0.042	0.814	0	0.71	0.943
BoPUB7	BoPUB43	0	0	0	0.51	0.51
BoPUB7	BoPUB50	0	0	0	0.508	0.508
BoPUB7	BoPUB51	0	0	0	0.461	0.461
BoPUB7	BoPUB52	0.043	0	0	0.516	0.517
BoPUB7	BoPUB54	0	0	0	0.461	0.461
BoPUB7	BoPUB58	0.045	0	0	0.531	0.532
BoPUB7	BoPUB60	0.043	0	0	0.516	0.517
BoPUB19	BoPUB32	0	0	0	0.701	0.701
BoPUB26	BoPUB23	0	0	0	0.578	0.578
BoPUB26	BoPUB36	0.056	0	0	0.593	0.599
BoPUB26	BoPUB42	0	0	0.8	0.916	0.802
BoPUB26	BoPUB45	0	0	0	0.57	0.57
BoPUB26	BoPUB58	0	0	0	0.578	0.578
BoPUB26	BoPUB64	0	0	0	0.411	0.41
BoPUB36	BoPUB23	0	0	0	0.475	0.475
BoPUB36	BoPUB42	0.056	0	0	0.593	0.599
BoPUB36	BoPUB58	0	0	0	0.475	0.475
BoPUB36	BoPUB64	0	0	0	0.573	0.573
BoPUB42	BoPUB23	0	0	0	0.578	0.578
BoPUB42	BoPUB45	0	0	0	0.57	0.57
BoPUB42	BoPUB58	0	0	0	0.578	0.578
BoPUB42	BoPUB64	0	0	0	0.411	0.41

The prediction of interactions between BoPUB proteins was performed by the STRING server. The values represent the accuracy probability of protein-protein interaction.

Table S6. Cis-regulatory elements in the promoters of *BoPUB* genes.

Cis-regulatory element	Number of gene	Function of cis-regulatory element	Type of cis-regulatory element
AACA_motif	BoPUB24	involved in endosperm-specific negative expression	Growth and development process
CAT-box	BoPUB3; BoPUB6; BoPUB10; BoPUB11; BoPUB14; BoPUB15; BoPUB18; BoPUB25; BoPUB28; BoPUB30; BoPUB31; BoPUB34; BoPUB38; BoPUB39; BoPUB43; BoPUB44; BoPUB45; BoPUB49; BoPUB50; BoPUB52; BoPUB53; BoPUB55; BoPUB56; BoPUB60; BoPUB62	cis-acting regulatory element related to meristem expression	
circadian	BoPUB1; BoPUB12; BoPUB15; BoPUB16; BoPUB17; BoPUB18; BoPUB23; BoPUB26; BoPUB32; BoPUB33; BoPUB38; BoPUB42; BoPUB46; BoPUB49; BoPUB54; BoPUB59; BoPUB63	cis-acting regulatory element involved in circadian control	
GCN4_motif	BoPUB5; BoPUB8; BoPUB15; BoPUB20; BoPUB21; BoPUB22; BoPUB38; BoPUB39; BoPUB40; BoPUB43; BoPUB48; BoPUB49; BoPUB50; BoPUB52; BoPUB55; BoPUB56; BoPUB57; BoPUB62; BoPUB65	cis-regulatory element involved in endosperm expression	
HD-Zip 1	BoPUB39; BoPUB57	element involved in differentiation of the palisade mesophyll cells	
MBSI	BoPUB8; BoPUB14; BoPUB21; BoPUB46; BoPUB47; BoPUB54	MYB binding site involved in flavonoid biosynthetic genes regulation	
MSA-like	BoPUB9; BoPUB11; BoPUB19; BoPUB27; BoPUB31; BoPUB43; BoPUB53	cis-acting element involved in cell cycle regulation	
O2-site	BoPUB6; BoPUB11; BoPUB13; BoPUB15; BoPUB19; BoPUB23; BoPUB24; BoPUB27; BoPUB30; BoPUB32; BoPUB33; BoPUB37; BoPUB38; BoPUB39; BoPUB49; BoPUB50; BoPUB52; BoPUB55; BoPUB58; BoPUB61	cis-acting regulatory element involved in zein metabolism regulation	
RY-element	BoPUB14; BoPUB32	cis-acting regulatory element involved in seed-specific regulation	
ABRE	BoPUB1; BoPUB2; BoPUB3; BoPUB4; BoPUB5; BoPUB6; BoPUB7; BoPUB8; BoPUB9; BoPUB10; BoPUB11; BoPUB12; BoPUB13; BoPUB14; BoPUB15; BoPUB16; BoPUB18; BoPUB19; BoPUB20; BoPUB21; BoPUB22; BoPUB23; BoPUB24; BoPUB25; BoPUB26; BoPUB27; BoPUB28; BoPUB29; BoPUB30; BoPUB31; BoPUB32; BoPUB33; BoPUB34; BoPUB35; BoPUB36; BoPUB37; BoPUB38; BoPUB39; BoPUB40; BoPUB41; BoPUB42; BoPUB43; BoPUB44; BoPUB45; BoPUB46; BoPUB47; BoPUB48; BoPUB49; BoPUB50; BoPUB51; BoPUB52; BoPUB53; BoPUB54; BoPUB55; BoPUB56; BoPUB57; BoPUB58; BoPUB59; BoPUB60; BoPUB61; BoPUB62; BoPUB65	cis-acting element involved in the abscisic acid responsiveness	Phytohormone response
AuxRR-core	BoPUB10; BoPUB27; BoPUB30; BoPUB38; BoPUB44; BoPUB45; BoPUB50; BoPUB53; BoPUB58; BoPUB65	cis-acting regulatory element involved in auxin responsiveness	
CGTCA-motif	BoPUB1; BoPUB2; BoPUB3; BoPUB4; BoPUB5; BoPUB6; BoPUB7; BoPUB8; BoPUB10; BoPUB11; BoPUB12; BoPUB13; BoPUB14; BoPUB15; BoPUB16; BoPUB17; BoPUB18; BoPUB19; BoPUB21; BoPUB22; BoPUB24; BoPUB25; BoPUB26; BoPUB27; BoPUB28; BoPUB29; BoPUB30; BoPUB31; BoPUB33; BoPUB34; BoPUB35; BoPUB36; BoPUB37; BoPUB38; BoPUB39; BoPUB41; BoPUB42; BoPUB43; BoPUB44; BoPUB45; BoPUB46; BoPUB47; BoPUB48; BoPUB49; BoPUB50; BoPUB51; BoPUB52; BoPUB53; BoPUB54; BoPUB55; BoPUB56; BoPUB58; BoPUB59; BoPUB60; BoPUB61; BoPUB62; BoPUB63; BoPUB64; BoPUB65	cis-acting regulatory element involved in the MeJA-responsiveness	
GARE-motif	BoPUB6; BoPUB7; BoPUB12; BoPUB17; BoPUB24; BoPUB27; BoPUB36; BoPUB37; BoPUB39; BoPUB42; BoPUB45; BoPUB49; BoPUB51; BoPUB58; BoPUB60; BoPUB62; BoPUB63; BoPUB65	gibberellin-responsive element	
P-box	BoPUB1; BoPUB2; BoPUB3; BoPUB4; BoPUB7; BoPUB11; BoPUB13; BoPUB15; BoPUB16; BoPUB20; BoPUB22; BoPUB27; BoPUB28; BoPUB34; BoPUB39; BoPUB40; BoPUB44; BoPUB47; BoPUB51; BoPUB52; BoPUB54; BoPUB56; BoPUB57; BoPUB59; BoPUB63	gibberellin-responsive element	
TATC-box	BoPUB3; BoPUB5; BoPUB9; BoPUB17; BoPUB23; BoPUB24; BoPUB33; BoPUB59; BoPUB61; BoPUB62; BoPUB64	cis-acting element involved in gibberellin-responsiveness	
TCA-element	BoPUB1; BoPUB2; BoPUB3; BoPUB4; BoPUB8; BoPUB9; BoPUB13; BoPUB15; BoPUB16; BoPUB17; BoPUB20; BoPUB21; BoPUB23; BoPUB25; BoPUB26; BoPUB28; BoPUB30; BoPUB31; BoPUB32; BoPUB33; BoPUB34; BoPUB36; BoPUB37; BoPUB40; BoPUB45; BoPUB47; BoPUB48; BoPUB49; BoPUB50; BoPUB51; BoPUB52; BoPUB54; BoPUB56; BoPUB58; BoPUB59; BoPUB60; BoPUB62; BoPUB65	cis-acting element involved in salicylic acid responsiveness	
TGACG-motif	BoPUB1; BoPUB2; BoPUB3; BoPUB4; BoPUB5; BoPUB6; BoPUB7; BoPUB8; BoPUB10; BoPUB11; BoPUB12; BoPUB13; BoPUB14; BoPUB15; BoPUB16; BoPUB17; BoPUB18; BoPUB19; BoPUB20; BoPUB21; BoPUB22; BoPUB24; BoPUB25; BoPUB26; BoPUB27; BoPUB28; BoPUB29; BoPUB30; BoPUB31; BoPUB33; BoPUB34; BoPUB35; BoPUB36; BoPUB37; BoPUB38; BoPUB39; BoPUB40; BoPUB41; BoPUB42; BoPUB43; BoPUB44; BoPUB45; BoPUB46; BoPUB47; BoPUB48; BoPUB49; BoPUB50; BoPUB51; BoPUB52; BoPUB53; BoPUB54; BoPUB55; BoPUB56; BoPUB58; BoPUB59; BoPUB60; BoPUB61; BoPUB62; BoPUB63; BoPUB64; BoPUB65	cis-acting regulatory element involved in the MeJA-responsiveness	
TGA-element	BoPUB1; BoPUB2; BoPUB4; BoPUB6; BoPUB7; BoPUB8; BoPUB11; BoPUB13; BoPUB19; BoPUB20; BoPUB21; BoPUB23; BoPUB24; BoPUB27; BoPUB29; BoPUB30; BoPUB31; BoPUB33; BoPUB37; BoPUB38; BoPUB43; BoPUB44; BoPUB48; BoPUB49; BoPUB50; BoPUB52; BoPUB54; BoPUB55; BoPUB58; BoPUB60; BoPUB61; BoPUB65	auxin-responsive element	
3-AF1 binding site	BoPUB4; BoPUB5; BoPUB28; BoPUB56	light responsive element	Light response
ACE	BoPUB1; BoPUB4; BoPUB6; BoPUB15; BoPUB16; BoPUB21; BoPUB22; BoPUB31; BoPUB32; BoPUB34; BoPUB41; BoPUB43; BoPUB44; BoPUB51; BoPUB54; BoPUB56; BoPUB61	cis-acting element involved in light responsiveness	
AE-box	BoPUB1; BoPUB2; BoPUB3; BoPUB5; BoPUB6; BoPUB8; BoPUB9; BoPUB10; BoPUB11; BoPUB12; BoPUB14; BoPUB15; BoPUB16; BoPUB17; BoPUB18; BoPUB22; BoPUB24; BoPUB25; BoPUB26; BoPUB28; BoPUB29; BoPUB30; BoPUB32; BoPUB34; BoPUB37; BoPUB38; BoPUB39; BoPUB40; BoPUB41; BoPUB42; BoPUB45; BoPUB46; BoPUB47; BoPUB48; BoPUB49; BoPUB51; BoPUB53; BoPUB54; BoPUB55; BoPUB58; BoPUB61; BoPUB63; BoPUB64	part of a module for light response	
ATC-motif	BoPUB4; BoPUB10; BoPUB23; BoPUB34; BoPUB40; BoPUB55; BoPUB60	part of a conserved DNA module involved in light responsiveness	
ATCT-motif	BoPUB9; BoPUB11; BoPUB19; BoPUB23; BoPUB25; BoPUB29; BoPUB32; BoPUB42; BoPUB43; BoPUB49; BoPUB50; BoPUB54; BoPUB61; BoPUB64	part of a conserved DNA module involved in light responsiveness	
Box 4	BoPUB1; BoPUB3; BoPUB4; BoPUB5; BoPUB7; BoPUB8; BoPUB9; BoPUB10; BoPUB11; BoPUB12; BoPUB14; BoPUB16; BoPUB18; BoPUB19; BoPUB20; BoPUB21; BoPUB22; BoPUB23; BoPUB24; BoPUB25; BoPUB26; BoPUB28; BoPUB30; BoPUB31; BoPUB32; BoPUB33; BoPUB35; BoPUB36; BoPUB37; BoPUB38; BoPUB39; BoPUB41; BoPUB42; BoPUB43; BoPUB44; BoPUB45; BoPUB47; BoPUB48; BoPUB49; BoPUB50; BoPUB51; BoPUB52; BoPUB53; BoPUB54; BoPUB58; BoPUB61; BoPUB62; BoPUB63; BoPUB64	part of a conserved DNA module involved in light responsiveness	
Box II	BoPUB20; BoPUB30; BoPUB37; BoPUB38; BoPUB46; BoPUB60; BoPUB63	part of a light responsive element	
chs-CMA1a	BoPUB9; BoPUB10; BoPUB12; BoPUB19; BoPUB23; BoPUB26; BoPUB28; BoPUB30; BoPUB31; BoPUB34; BoPUB35; BoPUB37; BoPUB39; BoPUB40; BoPUB41; BoPUB42; BoPUB44; BoPUB45; BoPUB49; BoPUB58; BoPUB60	part of a light responsive element	
chs-CMA2a	BoPUB1; BoPUB14; BoPUB34; BoPUB37	part of a light responsive element	
GA-motif	BoPUB3; BoPUB6; BoPUB13; BoPUB14; BoPUB18; BoPUB20; BoPUB30; BoPUB31; BoPUB39; BoPUB47; BoPUB56; BoPUB60	part of a light responsive element	
Gap-box	BoPUB42; BoPUB54	part of a light responsive element	
GATA-motif	BoPUB1; BoPUB2; BoPUB4; BoPUB9; BoPUB11; BoPUB12; BoPUB14; BoPUB16; BoPUB17; BoPUB18; BoPUB21; BoPUB23; BoPUB25; BoPUB26; BoPUB28; BoPUB30; BoPUB31; BoPUB32; BoPUB33; BoPUB34; BoPUB37; BoPUB38; BoPUB39; BoPUB42; BoPUB46; BoPUB49; BoPUB50; BoPUB51; BoPUB52; BoPUB54; BoPUB58; BoPUB60; BoPUB62; BoPUB64	part of a light responsive element	
G-Box	BoPUB1; BoPUB2; BoPUB3; BoPUB4; BoPUB6; BoPUB7; BoPUB8; BoPUB9; BoPUB10; BoPUB11; BoPUB12; BoPUB13; BoPUB14; BoPUB15; BoPUB16; BoPUB18; BoPUB19; BoPUB20; BoPUB21; BoPUB22; BoPUB23; BoPUB25; BoPUB26; BoPUB27; BoPUB28; BoPUB29; BoPUB30; BoPUB31; BoPUB32; BoPUB33; BoPUB34; BoPUB35; BoPUB36; BoPUB37; BoPUB38; BoPUB39; BoPUB40; BoPUB41; BoPUB42; BoPUB43; BoPUB44; BoPUB45; BoPUB46; BoPUB47; BoPUB48; BoPUB49; BoPUB50; BoPUB51; BoPUB52; BoPUB53; BoPUB54; BoPUB55; BoPUB56; BoPUB57; BoPUB58; BoPUB59; BoPUB60; BoPUB61; BoPUB62; BoPUB65	cis-acting regulatory element involved in light responsiveness	
GT1-motif	BoPUB3; BoPUB5; BoPUB7; BoPUB9; BoPUB10; BoPUB11; BoPUB12; BoPUB15; BoPUB19; BoPUB21; BoPUB22; BoPUB23; BoPUB24; BoPUB25; BoPUB26; BoPUB27; BoPUB28; BoPUB29; BoPUB32; BoPUB33; BoPUB34; BoPUB35; BoPUB37; BoPUB38; BoPUB39; BoPUB40; BoPUB43; BoPUB44; BoPUB45; BoPUB46; BoPUB47; BoPUB48; BoPUB49; BoPUB51; BoPUB52; BoPUB54; BoPUB56; BoPUB58; BoPUB59; BoPUB60; BoPUB61; BoPUB63; BoPUB64; BoPUB65	light responsive element	
I-box	BoPUB4; BoPUB9; BoPUB13; BoPUB21; BoPUB25; BoPUB28; BoPUB29; BoPUB31; BoPUB32; BoPUB33; BoPUB34; BoPUB37; BoPUB40; BoPUB43; BoPUB44; BoPUB49; BoPUB50; BoPUB55; BoPUB56; BoPUB58; BoPUB65	part of a light responsive element	
LAMP-element	BoPUB1; BoPUB11; BoPUB13; BoPUB20; BoPUB28; BoPUB30; BoPUB38; BoPUB40; BoPUB62	part of a light responsive element	
MRE	BoPUB3; BoPUB7; BoPUB11; BoPUB13; BoPUB14; BoPUB16; BoPUB23; BoPUB27; BoPUB28; BoPUB34; BoPUB35; BoPUB36; BoPUB38; BoPUB43; BoPUB48; BoPUB51; BoPUB59; BoPUB64	MYB binding site involved in light responsiveness	
Sp1	BoPUB1; BoPUB4; BoPUB8; BoPUB12; BoPUB22; BoPUB25; BoPUB33; BoPUB34; BoPUB39; BoPUB52; BoPUB53; BoPUB58; BoPUB61; BoPUB65	light responsive element	
TCCC-motif	BoPUB1; BoPUB3; BoPUB7; BoPUB8; BoPUB11; BoPUB14; BoPUB21; BoPUB25; BoPUB27; BoPUB28; BoPUB31; BoPUB37; BoPUB38; BoPUB45; BoPUB49; BoPUB52; BoPUB59; BoPUB60; BoPUB62; BoPUB63; BoPUB65	part of a light responsive element	
TCT-motif	BoPUB1; BoPUB2; BoPUB4; BoPUB5; BoPUB6; BoPUB8; BoPUB9; BoPUB11; BoPUB13; BoPUB14; BoPUB16; BoPUB18; BoPUB20; BoPUB21; BoPUB24; BoPUB25; BoPUB27; BoPUB28; BoPUB29; BoPUB32; BoPUB33; BoPUB34; BoPUB36; BoPUB37; BoPUB39; BoPUB40; BoPUB41; BoPUB44; BoPUB45; BoPUB46; BoPUB51; BoPUB52; BoPUB54; BoPUB55; BoPUB56; BoPUB58; BoPUB59; BoPUB61; BoPUB64; BoPUB65	part of a light responsive element	

ARE	BoPUB1; BoPUB2; BoPUB3; BoPUB4; BoPUB5; BoPUB6; BoPUB7; BoPUB8; BoPUB9; BoPUB10; BoPUB11; BoPUB12; BoPUB13; BoPUB14; BoPUB15; BoPUB16; BoPUB17; BoPUB18; BoPUB19; BoPUB20; BoPUB21; BoPUB22; BoPUB23; BoPUB24; BoPUB25; BoPUB26; BoPUB27; BoPUB28; BoPUB29; BoPUB30; BoPUB31; BoPUB32; BoPUB33; BoPUB34; BoPUB35; BoPUB36; BoPUB37; BoPUB38; BoPUB39; BoPUB40; BoPUB41; BoPUB42; BoPUB43; BoPUB44; BoPUB45; BoPUB46; BoPUB47; BoPUB48; BoPUB49; BoPUB50; BoPUB51; BoPUB52; BoPUB53; BoPUB54; BoPUB55; BoPUB56; BoPUB57; BoPUB58; BoPUB59; BoPUB60; BoPUB61; BoPUB62; BoPUB63; BoPUB64; BoPUB65	cis-acting regulatory element essential for the anaerobic induction	Stress response
GC-motif	BoPUB6; BoPUB11; BoPUB22; BoPUB25; BoPUB34; BoPUB44; BoPUB45; BoPUB50; BoPUB56; BoPUB58; BoPUB65	enhancer-like element involved in anoxic specific inducibility	
LTR	BoPUB3; BoPUB5; BoPUB6; BoPUB8; BoPUB11; BoPUB12; BoPUB13; BoPUB14; BoPUB15; BoPUB16; BoPUB18; BoPUB19; BoPUB20; BoPUB22; BoPUB23; BoPUB24; BoPUB25; BoPUB27; BoPUB29; BoPUB31; BoPUB34; BoPUB37; BoPUB38; BoPUB40; BoPUB42; BoPUB44; BoPUB45; BoPUB47; BoPUB48; BoPUB49; BoPUB50; BoPUB53; BoPUB55; BoPUB57; BoPUB58; BoPUB60; BoPUB61; BoPUB63	cis-acting element involved in low-temperature responsiveness	
MBS	BoPUB1; BoPUB4; BoPUB5; BoPUB6; BoPUB7; BoPUB8; BoPUB10; BoPUB12; BoPUB13; BoPUB14; BoPUB15; BoPUB16; BoPUB17; BoPUB21; BoPUB22; BoPUB24; BoPUB25; BoPUB26; BoPUB27; BoPUB29; BoPUB30; BoPUB31; BoPUB32; BoPUB34; BoPUB35; BoPUB36; BoPUB37; BoPUB38; BoPUB39; BoPUB40; BoPUB41; BoPUB42; BoPUB43; BoPUB44; BoPUB45; BoPUB46; BoPUB47; BoPUB48; BoPUB50; BoPUB51; BoPUB52; BoPUB53; BoPUB54; BoPUB56; BoPUB57; BoPUB58; BoPUB62; BoPUB63; BoPUB65	MYB binding site involved in drought-inducibility	
TC-rich repeats	BoPUB1; BoPUB3; BoPUB4; BoPUB5; BoPUB6; BoPUB9; BoPUB11; BoPUB12; BoPUB16; BoPUB17; BoPUB18; BoPUB32; BoPUB33; BoPUB34; BoPUB37; BoPUB39; BoPUB40; BoPUB44; BoPUB45; BoPUB46; BoPUB47; BoPUB50; BoPUB51; BoPUB52; BoPUB54; BoPUB56; BoPUB58; BoPUB64; BoPUB65	cis-acting element involved in defense and stress responsiveness	

Table S7. Primers used for qRT-PCR analysis.

Gene ID[a]	Primer name	Primer sequence (5'-3')
Bol005722	BoPUB3-F	TGCGGTTGAACTTGGTTTGG
	BoPUB3-R	ACAACTCAGCTGAACGTTGC
Bol007667	BoPUB6-F	TCGTCTGCGAAAACGCTAAC
	BoPUB6-R	TGAGCGCTTTGTTCGAATCC
Bol007668	BoPUB7-F	TTGAGCGCGCTTCTCAATTG
	BoPUB7-R	GAAGAGACTCCGAAGTTCAAGC
Bol008036	BoPUB8-F	ACAGCTTAGTGCAGACGAAC
	BoPUB8-R	TTGCGCCTCTTTTGATCCTG
Bol008152	BoPUB9-F	TGGTGAATCCGTTGTTGAGC
	BoPUB9-R	ACGTTAGGGTTTTCGCAGAC
Bol008441	BoPUB10-F	TGTCTCGAAGATGCAAAGCC
	BoPUB10-R	AGGGCATCGACTTTGCATTG
Bol008579	BoPUB11-F	CGTGCCTAATCGAGCTTTGAAG
	BoPUB11-R	TTCAACCGCAGCTTTTGTCG
Bol010460	BoPUB15-F	ACAACACAGCAAGAGCAAGC
	BoPUB15-R	ACCTTCTTGTAGCACCATCTGG
Bol015885	BoPUB19-F	TAACGGTCGAGATCGGTTAACG
	BoPUB19-R	AACCGGCGCAATCAATCTTC
Bol021972	BoPUB28-F	TGAGGGTTACTGAGAGTTGCAC
	BoPUB28-R	ATCACGAGCAACAGCTTTGC
Bol022718	BoPUB29-F	AAGAGCTGCACAGGAAAACC
	BoPUB29-R	AACGCTTTCGCTGCTTCTAC
Bol023451	BoPUB31-F	AGCCAAGCTTCCGCAATTTC
	BoPUB31-R	AGAATCTCCCTCGCGTTATGAC
Bol023972	BoPUB32-F	TTGGTTAGCGCCTTTCATCG
	BoPUB32-R	TTGGCATCTTGTCCAAACGC
Bol025043	BoPUB34-F	TGAAGTTGCGGATCCAATGC
	BoPUB34-R	GCATTGCTGATTTCGTTGCC
Bol033248	BoPUB45-F	TGGTGCAGTTGTGTTCATGG
	BoPUB45-R	AAGTAACTGTGCCGCTTTGC
Bol034488	BoPUB46-F	TCAGATACGGTTGGAGTGGTTG
	BoPUB46-R	AATGGCTTTGGTGCAGTTGC
Bol034491	BoPUB48-F	TGATTGCGCAATGGTGTCAG
	BoPUB48-R	TCGCTGAACAATCCAATGGG
Bol036718	BoPUB50-F	AACATCACGTGCCCCAAAAC

	BoPUB50-R	TTGCTCTGCAGTGCTTTTGG
Bol039769	BoPUB54-F	ACAAACGGTGTTTGGAGGTG
	BoPUB54-R	AGAGTCAAACCCGTTGTTGC
Bol040675	BoPUB56-F	ATGCAGAATCCCCATGTTGC
	BoPUB56-R	AGTCATCGGCGATTTTTCACC
Bol041392	BoPUB58-F	AACAGCGAGGACCAGAAAGTAG
	BoPUB58-R	TTGGGCAGCGAAAATCATCG
Bol041756	BoPUB59-F	AGCCGTGTTACTAAACCTAGCC
	BoPUB59-R	ATCACAAGGCAAGCAACAGC
Bol041877	BoPUB60-F	TGCTGCAGACGCTTTTGTTC
	BoPUB60-R	TCTGGAAAACACGAGCAAGC
Bol044051	BoPUB61-F	AACGACAGTGGTGGATCAGAC
	BoPUB61-R	TTGTTCATGCCATCGCCTTC
Bol044176	BoPUB62-F	TCAGCAGCAACGTTGTTCAG
	BoPUB62-R	AACAAAGCAGACGCAGCATC
Bol044185	BoPUB63-F	TGCTCGCATCGATCATTTGG
	BoPUB63-R	AATACGACATCCGCAGCATG
Bol045811	BoPUB65-F	TCATCGTTAACCGCATGCAC
	BoPUB65-R	AATCACCGGATCAGTCATGACC

[a]Accession number from the *Brassica oleracea* var. *capitata*_446_v1.0 genome database.

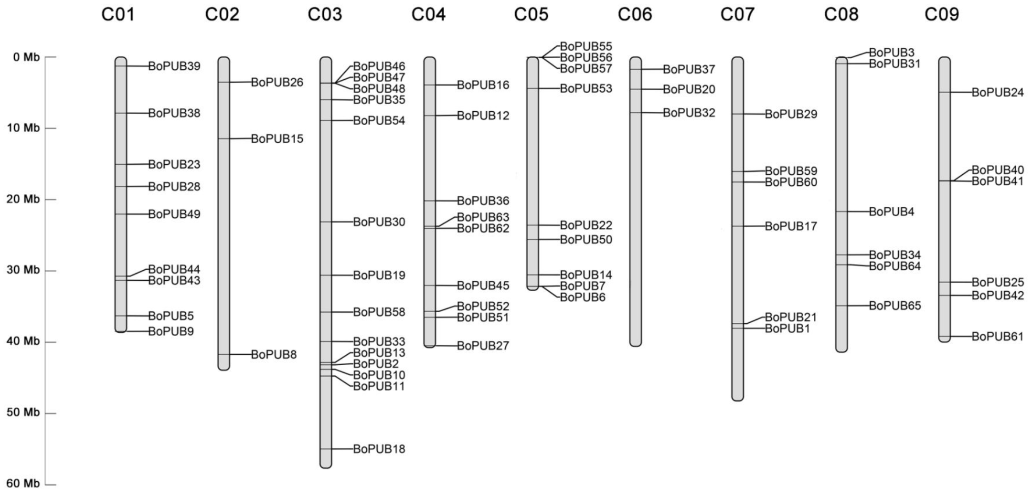

Figure S1. Chromosomal localization of *BoPUB* genes.

Nine chromosomes (C01-09) in *Brassica oleracea* are arranged in a parallel pattern, and the location of 65 *BoPUB* genes is marked.

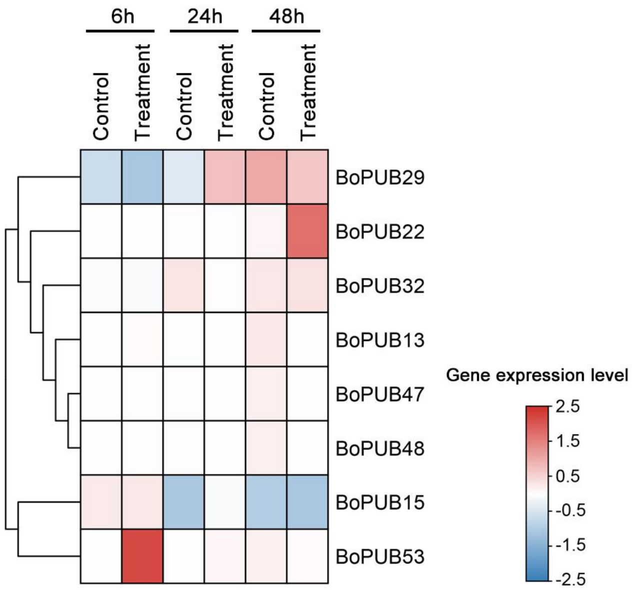

Figure S2. Expression analysis of *BoPUB* genes after *Xanthomonas campestris* pv. *campestris* infection.

The expression data of *BoPUB* genes were obtained from the microarray gene expression data of cabbage seedlings at 6 h, 24 h, and 48 h after treatment by *Xanthomonas campestris* pv. *campestris*. The expression levels of *BoPUB* genes in control samples (Control) and pathogen-infected samples (Treatment) were plotted in a color heatmap. Each row and column in the color heatmap indicate a single gene and time point, respectively, and their names are shown.
